# An Overview of the Nano-Enhanced Phase Change Materials for Energy Harvesting and Conversion

**DOI:** 10.3390/molecules28155763

**Published:** 2023-07-30

**Authors:** José Pereira, Ana Moita, António Moreira

**Affiliations:** IN+ Center for Innovation, Technology and Policy Research, Instituto Superior Técnico, Universidade de Lisboa, Av. Rovisco Pais, 1049-001 Lisbon, Portugal; anamoita@tecnico.ulisboa.pt (A.M.); aluismoreira@tecnico.ulisboa.pt (A.M.)

**Keywords:** heat transfer, PV systems, energy conversion, phase change materials

## Abstract

This review offers a critical survey of the published studies concerning nano-enhanced phase change materials to be applied in energy harvesting and conversion. Also, the main thermophysical characteristics of nano-enhanced phase change materials are discussed in detail. In addition, we carried out an analysis of the thermophysical properties of these types of materials as well as of some specific characteristics like the phase change duration and the phase change temperature. Moreover, the fundamental improving techniques for the phase change materials for solar thermal applications are described in detail, including the use of nano-enhanced phase change materials, foam skeleton-reinforced phase change materials, phase change materials with extended surfaces, and the inclusion of high-thermal-conductivity nanoparticles in nano-enhanced phase change materials, among others. Those improvement techniques can increase the thermal conductivity of the systems by up to 100%. Furthermore, it is also reported that the exploration of phase change materials enhances the overall efficiency of solar thermal energy storage systems and photovoltaic-nano-enhanced phase change materials systems. Finally, the main limitations and guidelines for future research in the field of nano-enhanced phase change materials are summarized.

## 1. Introduction

**Figure 1:**
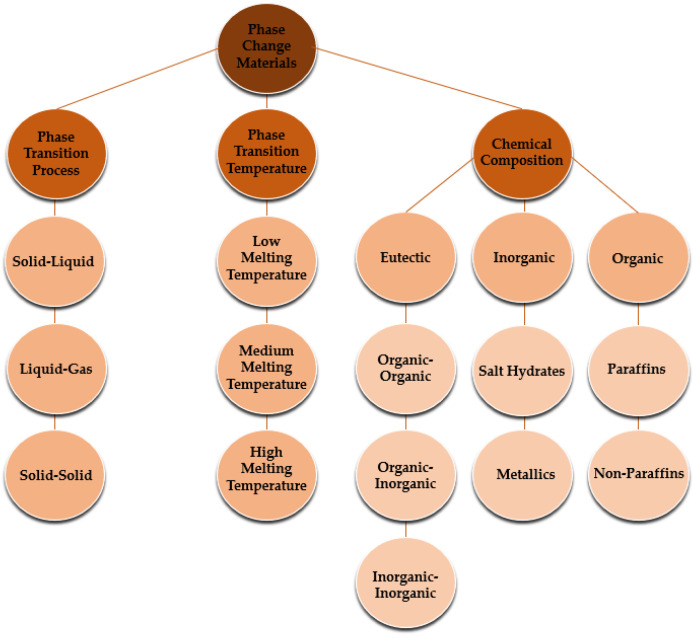
Fundamental criteria and types of phase change materials.

Nanomaterials have been intensively studied in recent years, given that they possess great potential for technical breakthroughs and offer beneficial features for the use of nanomaterials in diverse applications. Nanotechnology can be of relevance in distinct technological areas of actuation, including IT technologies, medicine, and innovative materials, among others. Particularly, energy storage and conversion are among the most challenging nanotechnological areas, given that the use of nanomaterials may give way to remarkable advances in many applications. The use of nanoparticles and other nanostructures has been evaluated in-depth in the past few decades because of the great number of possible applications. The fundamental direction of this overview is focused on energy harvesting and conversion. Furthermore, the use of nanofluids in solar absorption thermal management was presented by the authors Nourafkan et al. [1]. The authors emphasized the experimental photo-thermal conversion efficiency using a solar simulator. The research team added lithium bromide nanoparticles at a weight fraction of 50% wt. to water. The obtained experimental results indicated that the incorporation of the nanoparticles enhanced the light trapping efficiency and hence augmented the bulk temperature. The verified efficiency increase was reported to be between nearly 5% and 12%, and the authors concluded that the inclusion of the nanoparticles was very suitable for solar absorption cooling ends. Also, the possible application of nano-improved materials in thermoelectric generation purposes was inferred by the authors Raihan et al. [2]. A nanostructured bulk alloy at 0.1% vol. of silicon carbide nanoparticles was employed with respect to the examination of the heat conduction and heat transfer loss into the surroundings. The authors confirmed that the addition of nanoparticles increased the thermal efficiency in the range of 7.3% to 8.7%. Moreover, an evacuated tube solar collector system having a parabolic concentrator and using graphene nanofluids was studied by the authors Natividade et al. [3]. The focus of their research was to evaluate the thermal efficiency of the system. The obtained results showed that the thermal efficiency of the solar collector was enhanced by between 31% and 76% compared to that achieved with the base fluid. The inclusion of nanoparticles for photovoltaic purposes was studied by the researchers Salem et al. [4], who analyzed the cooling effect on photovoltaics with the use of phase change materials. In their experimental work, an alumina/phase change material mixture having diverse concentrations of nanoparticles was added to water. The effect of the occupation ratio in the channels was evaluated, and from the obtained results, the authors revealed that it was possible to achieve an increase in the delivered power output from the photovoltaic panel, and a nanoparticle fraction of 1% wt. maximized the increase in the power output. The impact of the addition of silica and alumina nanoparticles in low-salinity hot water was addressed by the authors Ding et al. [5]. The goal of the study was to infer the potential to enhance the heavy oil recovery process. The numerical simulations showed an increase in the heavy oil recovery rate by between 2.4% and 7.2% with the inclusion of nanoparticles compared to the recovery rate of conventional water injection. The authors also reported that it would be possible to enhance the oil recovery by about 40%. Furthermore, an innovative thermomechanical fluid composed of nickel–bromine ionic liquid with the addition of vanadium dioxide nanoparticles was developed by the researchers Chen et al. [6] to produce an innovative composite film. The incorporation of the nanoparticles provided a 27% increase in the ultrahigh optical properties compared to the nearly 14% increase provided by vanadium dioxide film alone. This type of film could be an effective thermochromic material for smart window general ends. Also, the obtained results could provide thermal efficiency enhancements in residential buildings. Experimental work on a mineral lubricant with the addition of titanium oxide nanoparticles was carried out by the authors Adelekan et al. [7] using a liquefied petroleum gas refrigerant. The results showed that the nanoparticles in the mineral oil enhanced the overall efficiency of the refrigerator. Also, the results indicated that the engaged power was reduced from 8% to around 15%, depending on the concentration of the nanoparticles. The inclusion of nanoparticles of silica in fatty acids was elaborated by the authors Martín et al. [8] to create a potentially effective nano-enhanced phase change material having enhanced thermal conductivity, which is adequate for building thermal management applications. The researchers found that the addition of nanoparticles increased the thermal conductivity and heat transfer rate. The evaluation of a phase change material melting rate for thermal energy storage purposes was reported by the researchers Ren et al. [9], who considered the addition of nanoparticles. The obtained results showed that the energy storage efficiency decreased with an increasing concentration of nanoparticles. The reasons behind such a result were the augmented dynamic viscosity of the phase change material together with the poor energy storage capability. Also, one study of the direct absorption collectors using thermal fluids enhanced with nanoparticles was published by the researchers Balakin et al. [10]. For this purpose, they performed a CFD analysis, and the fundamental outcome was that the incorporation of certain nanoparticles caused an efficiency enhancement of the absorption collector of approximately 10%. In addition, the highest enhancement in the collector thermal performance was found when a magnetic nanofluid was considered, with an efficiency increase of nearly 30%. The inclusion of copper oxide and aluminum oxide nanoparticles and carbon nanotubes in water was numerically investigated by the authors Bonab and Javani [11] for a solar collector having absorbing pipes. The performance of the solar collector was enhanced with the incorporation of nanoparticles as compared to that of the water itself. The highest efficiency enhancement was found at concentrations less than 5% vol. The inclusion of nanoparticles for the amelioration of green hydrogen production rates by dark fermentation was published by the researchers Kumar et al. [12]. Reviews on the nanoparticles of silver, iron, titanium oxide, nickel, and others were obtained, and in all cases, the production rate of the eco-friendly hydrogen was enhanced. A numerical performance analysis of the gallium arsenide plasmonic solar cells with the incorporation of silver and gold nanoparticles was described by the researchers Zhang et al. [13]. The researchers reported that the inclusion of the nanoparticles enhanced the light absorption of gallium arsenide solar cells. The highest enhancement of the optical–electrical conversion rate was attained with the gold nanoparticles. It was also highlighted by the authors that with the optimization of the nanoparticle properties, it was possible to diminish the thermalization losses and ameliorate the electrical overall performance. The diversified factors affecting the thermal performance of flat-plate solar collectors were addressed by the researchers Zayed et al. [14] using nanofluids. The results indicated that the most adequate nanoparticles from the energy and exergy standpoints were the carbon-based ones. The efficiency enhancement ranged from around 6% to nearly 37% compared to those obtained with the traditionally employed nanofluids, at weight fractions of up to 2% wt. An analytical study was carried out by the researchers Abdelrazik et al. [15] to evaluate the thermal performance improvement in hybrid photovoltaic–thermal solar collectors. The chosen nanoparticles were included above the photovoltaic module and on the backside of the photovoltaic panel with a phase change material layer. The performed numerical analysis demonstrated that the performance of the examined photovoltaic/thermal system decreased by approximately 6.7% with the incorporation of the nanoparticles. Nonetheless, when considering an optical filtration above the photovoltaic panel, the efficiency of the system was enhanced from 6% to 12%. Innovative emulsions of paraffin and water mixtures were evaluated by the authors Agresti et al. [16] at concentrations between 2% wt. and 10% wt. The results indicated that the melting heat was decreased compared to that achieved with the base fluid alone and the thermal capacity was enhanced by up to 40%. An overview of heat transfer improvement was published by the researchers Rehman et al. [17]. The phase change materials composed of metallic and carbon porous materials or foams were the focus of the survey. The research team reported that the incorporation of nanoparticles in porous materials or foams can improve the thermal conductivity by between 3 and 500 times. Some useful correlations of thermal conductivity were also highlighted along with the assessed research gaps. The addition of inorganic nanoparticles in the phase change materials was studied by the researchers Sheikholeslami and Mahian [18] for energy storage applications. The authors also applied an external magnetic field, and the results indicated that the addition of nanoparticles at concentrations up to 4% wt. provided a 14% decrease in the solidification time. The combined application of the magnetic field and nanoparticles turned out to be a very suitable option to drastically augment the solidification for energy storage purposes. The diagram in Figure 1 summarizes the main criteria and types of the phase change materials. In sum, this review intends to comprehensively discuss the impact of adding nanoparticles to base phase change materials’ thermophysical characteristics like thermal conductivity, specific heat capacity, viscosity, density, latent heat, subcooling, phase change duration, and phase change temperature. Also, the eventual alterations in the thermophysical properties of the nano-enhanced phase change materials after being subjected to continuous thermal cycling are among the main objectives of this work. Additionally, the ideal characteristics of the nano-enhanced phase change materials, thermal-performance-influencing factors, potential applications, limitations, challenges, and future research routes regarding nano-enhanced phase change materials will be addressed in detail. 

**Figure 2:**
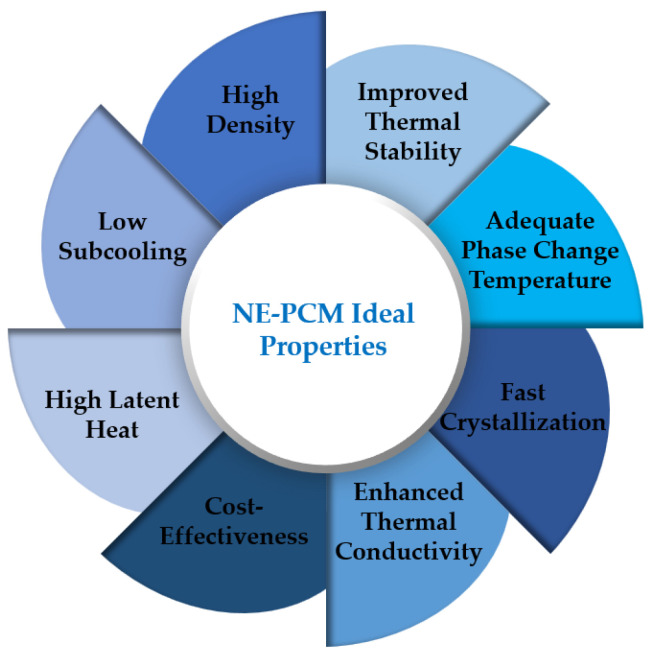
Main characteristics of an ideal nano-enhanced phase change material.

This review mostly deals with experimental and numerical research of nano-enhanced phase change materials. A similar overview was performed by the researchers Punniakodi and Senthil [19]. However, these authors focused on research studies dealing with the solar thermal energy storage applications of phase change materials with the incorporation of nano-scaled enhancers. Moreover, the rigid separation between nanofluids and nanocomposites can be considered outdated because of the wider field of application of nanomaterials in this context. Many nanofluids can be used as nano-enhanced phase change materials and vice versa, depending on the material phase. An example of this can be water improved with the addition of nanostructures or a paraffin nanofluid. Some researchers have already incorporated both nanofluids and nanocomposites in their review articles. Consequently, this review also investigates both types of materials. In this sense, a review was carried out by the authors Kibria et al. [20], focusing on the study of the thermophysical properties of nano-enhanced phase change materials, nanocomposites, and additives. A similar overview was published by the authors Leong et al. [21], in which they comprehensively addressed the thermal conductivity and latent heat capacity of nano-enhanced phase change materials along with their main potential applications. Their focus was on low-temperature energy storage applications between 20 °C and 37 °C. Accordingly, the authors Teggar et al. [22] published an overview paper concerning the available works on nano-enhanced phase change materials, their synthesis methodologies, thermophysical characteristics, and overall cost. The authors also analyzed the latent heat, specific heat, and thermal conductivity of these materials. The research presented herein addresses the less-investigated potential applications, pinpointing further research routes and attempting to fill the existing research gaps. This paper presents guidelines for researchers dealing with enhanced materials while giving insights into their application in diverse engineering systems. The main objective of the work was to pinpoint the potential future nano-enhanced phase change materials applications and to highlight the relevance of their preparation attained advances in line with the application of these enhanced materials. When considering nano-enhanced phase change materials for possible applications, the research community should be aware of their preparation challenges and improvements in their thermophysical properties. Figure 2 shows the fundamental enhanced properties that an optimal nano-enhanced phase change material should have.

## 2. Preparation Methods of Nano-Enhanced Phase Change Materials

**Figure 3:**
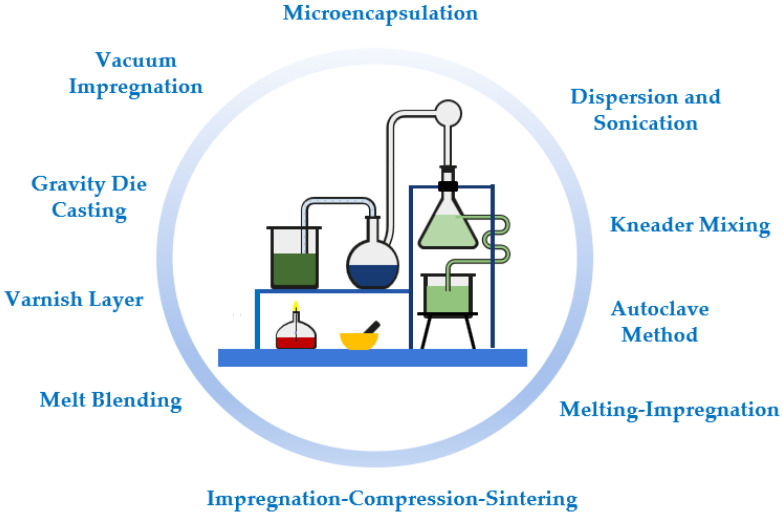
Main preparation methods of phase change materials and nano-enhanced phase change materials.

It should be noted that most of the published research studies describe their preparation methods of nano-enhanced phase change materials in an incomplete manner since important details are usually missing. Such details are often the amounts included in the final formulation, stirring revolutions, temperature range, duration and sonication power and frequency, manufacturer and model of the employed equipment, and manufacturer of the employed nanomaterials. These absent parameters are vital for a proper synthesis method and are very useful for the reproducibility of the experiments and the corresponding repeatability of results. Nonetheless, it can be stated that the main literature findings showed that diverse parameters affect the thermophysical characteristics of the final nano-enhanced phase change materials. The morphology of the added nanoparticles was found to be critical, along with the sonication stage. The following sections will briefly describe some of the synthesis routes followed. Figure 3 presents the main preparation methods of phase change materials and nano-enhanced phase change materials.

### 2.1. Microencapsulation Methods

**Figure 4:**
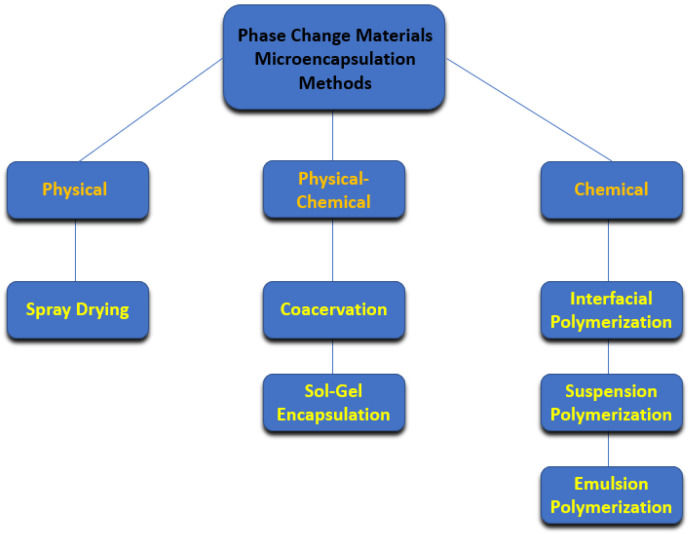
Main microencapsulation phase change materials’ preparation methods.

Encapsulated phase change materials were discussed by the authors De Matteis et al. [23] as an effective option for residential building thermal management applications. The researchers confirmed that the use of a nano-encapsulated phase change material can reduce the heating/cooling demands in residential buildings by between 1% and nearly 4%. Figure 4 summarizes the main microencapsulation methods for producing phase change materials.

The microencapsulated phase change materials are produced using physical, physical–chemical, and chemical methodologies. Among the physical ones, only the spray-drying technique can be used to produce microencapsulated phase change materials. Techniques like air suspension coating are not entirely adequate for the encapsulation of phase change materials [24]. Other physical methods including centrifugal extrusion, vibrational nozzle, and solvent evaporation have some potential and should be more profoundly investigated. The spray-drying technique is cost-effective and enables the preparation of microencapsulated phase change materials, which can be easily applied and scaled. Nonetheless, researchers should be aware of the eventual agglomeration that can occur during this preparation approach. The sol–gel and coacervation methods are the most employed physical–chemical methods in the fabrication of microencapsulated phase change materials. The microencapsulation of phase change materials using inorganic shell materials can be performed through the sol–gel method. An alternative synthesis route includes chemical methods that encompass interfacial, suspension, and emulsion polymerization techniques. Chemical methods are the most employed in the microencapsulation of phase change materials using a broad variety of shell materials. Moreover, it can be noted that some advantageous characteristics of microencapsulated phase change materials are their improved mechanical and chemical stability, extended thermal cycling, and prevention of chemical reactions with other materials. The main limitations of this type of phase change material are their high cost, the relative decrease in thermal conductivity, and leakage risk.

### 2.2. Dispersion and Sonication

**Figure 5:**
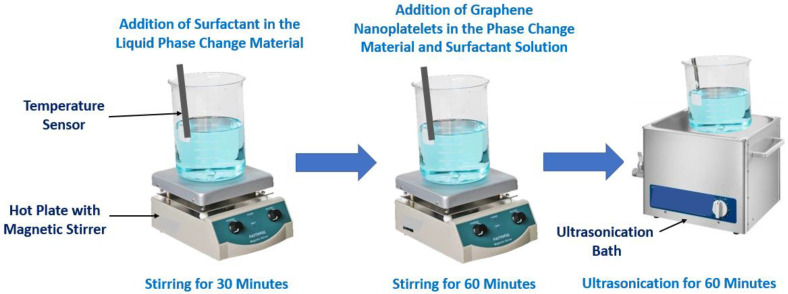
Schematic illustration of a typical preparation method of a nano-enhanced phase change material composed of a liquid base phase change material with the addition of graphene nanoparticles.

The researchers Liu and Yang [25] analyzed titanium oxide nanoparticles dispersed in inorganic hydrated salt phase change materials composed of disodium hydrogen phosphate dodecahydrate and sodium carbonate decahydrate. The solution had a mass ratio of 60:40 and the concentrations of the nanoparticles ranged between 0.1% wt. and 0.5% wt. The solution was first placed inside a beaker in a thermostatic water bath at 55 °C. After this step, the nano-enhanced phase change material was stirred for two hours at 1500 rpm, followed by ultrasonication for 30 min at 120 W. Moreover, the researchers Munyalo et al. [26] synthesized an aqueous solution having 24% wt. of BaCl_2_⋅2H_2_O with the incorporation of magnesium oxide nanoparticles and multi-walled carbon nanotubes at weight concentrations between 0.2% wt. and 1% wt. The enhanced solutions were mixed in a magnetic stirrer and ultrasonicated for 45 min at 50 °C. Nonetheless, the authors did not report the temperature and revolutions of the stirring procedure followed. On the other hand, the researchers Wang et al. [27] evaluated the thermophysical characteristics of paraffin with the addition of alumina nanoparticles. Alumina nanoparticles at volume concentrations of 0.001% vol., 0.005% vol., and 0.01% vol. were incorporated into the paraffin and dispersed using 30 min of magnetic stirring and one and a half hours of an ultrasonication procedure. The utilized equipment and other experimental parameters were not specified by the authors. The CTAB surfactant was added to avoid the possible agglomeration of nanoparticles. The published scientific articles reported that the nano-enhanced phase change materials are usually composed of paraffin wax and other organic and inorganic phase change materials to which diverse nanomaterials are added. Also, the authors Warzoha et al. [28] evaluated the temperature dependence on the thermal properties of paraffin with the addition of herringbone-style graphite nanofibers at volumetric concentrations between 0.05% vol. and 11.4% vol. A hot plate was employed to melt the phase change material at 80 °C, and to disperse the nanofibers, a sonicator was used for two hours at a frequency of 32 Hz. Moreover, the authors Nourani et al. [29] dispersed alumina nanoparticles at different concentrations from 2.5% wt. to 10% wt. in paraffin wax with the aid of a surfactant. The nanoparticles were mixed for one hour with the phase change material employing a magnetic stirrer and a vacuum pump at 600 mm Hg vacuum. After that, the ultrasonic vibration was used with an operating power of 80 W and a frequency of 50 kHz for two hours. During the mixing, the solvent petroleum ether was added to maintain the liquid phase which was then removed by heating the mixture. In addition, the researchers Motahar et al. [30] carried out an analysis study on the solidification of n-octadecane phase change material with the addition of titanium oxide nanoparticles at concentrations from 1.0% wt. to 4.0% wt. The blend was first mechanically stirred and then placed in an ultrasonic bath at 40 °C for 15 min. Moreover, the authors Wang et al. [31] evaluated the influence of the addition of graphite nanoparticles at 0.05% wt. and 0.1% wt. on the photothermal behavior and thermophysical features of a water/paraffin emulsion. The nanoparticles were dispersed with a rotor–stator homogenizer for five minutes at 12,000 rpm. Nonetheless, the investigation team did not specify the temperature of the homogenization of the mixture. The authors Chieruzzi et al. [32] fabricated a nitrate salt eutectic mixture of 60% wt. NaNO_3_ and 40% wt. KNO_3_ with 1% wt. of alumina and silica nanoparticles. The authors used a twin-screw micro-compounder to mix the material at high temperatures and evaluated the efficiency of two different settings for the experimental parameters, one for 15 min at 100 rpm and the other for 30 min at 200 rpm. The authors reported that the use of 200 rpm for 30 min was the best mixing strategy. In addition, the researchers Ebadi et al. [33] dispersed copper oxide nanoparticles at concentrations of 0.1% wt. and 1% wt. in coconut oil and examined the melting process and thermophysical properties. To achieve a uniform dispersion of the nanoparticles, a magnetic stirrer was used at 60 °C for 12 h. After that, the synthesis was followed by 30 min of ultrasonication. The temperature and frequency of the ultrasonication stage and the rotations per minute imposed in the magnetic stirrer were not reported by the authors. In addition, the researchers Salian and Suresh [34] investigated the impact of the addition of copper oxide nanoparticles at concentrations between 0.1% wt. and 0.5% wt. on the thermophysical characteristics of D-Mannitol. To uniformly disperse the nanoparticles in the phase change material, a ball mill was employed for two and a half hours at 250 rpm, and after that, the preparation method was resumed with the use of a sonicator at a frequency of 40 kHz. The authors did not specify the temperature and time of the ultrasonication procedure. Moreover, the researchers Praveen and Suresh [35] studied the heat transfer behavior of neopentyl glycol and copper oxide nanoparticles at concentrations between 0.5% wt. and 3.0% wt. Neopentyl glycol with the addition of nanoparticles was produced with the help of a ball mill at 200 rpm for two hours, followed by an ultrasonication stage using a probe sonicator at 40 kHz for 10 min. However, the researchers did not specify the temperature values of the synthesis methodology. Figure 5 schematically represents a typical preparation method of a nano-enhanced phase change material with the addition of graphene nanoparticles. 

### 2.3. Autoclave Method

The researchers Zeng et al. [36] followed the autoclave method to synthesize a nano-enhanced phase change material and chemically intercalated flake graphite to produce expanded graphite. The expanded graphite was then expanded by means of microwave irradiation to obtain expanded graphite. The tetradecanol/expandable graphite nano-enhanced phase change material was produced by mixing tetradecanol with expanded graphite through an autoclave method. The nano-enhanced phase change material produced exhibited excellent thermal energy storage capacity. In detail, the expanded graphite was prepared with a certain amount of natural flake graphite added to a solution of glacial acetic acid and nitric acid under stirring in a 35 °C water bath to form a uniformly distributed mixture. Then, potassium permanganate was added to the mixture with continuous stirring. During the stirring stage, a nitric acid/acetic acid/graphite intercalation compound was formed. Then, this intercalation compound was filtered, washed, and dried at 50 °C under vacuum to obtain dry expanded graphite. The obtained expanded graphite was then radiated for 1 min in a microwave oven to obtain the expanded graphite. The expansion ratio of expanded graphite was measured with the following procedure: 0.20 g expandable graphite in a 50 mL beaker was irradiated for one minute, and then the volume of the obtained expanded graphite was recorded. The obtained mean volume value divided by 0.20 g resulted in the expansion ratio of the expanded graphite of mL/g. After that, the expandable graphite, tetradecanol, and anhydrous ethanol were mixed in an autoclave, which was heated at 120 °C for one day and then cooled to room temperature. The mixture was then heated at 50 °C under reduced pressure to evaporate the ethanol. After that, the mixture was dried under vacuum for one day to obtain the tetradecanol/expandable graphite nano-enhanced phase change material. 

### 2.4. Gravity Die Casting

Gravity die casting is a technique that was employed by the researchers Mandal et al. [37], who reported the effects of using the phase change material paraffin with the addition of copper oxide nanoparticles as a storage medium on the performance of a solar water heater. The authors found that with increasing copper oxide nanoparticles, the temperature of the nano-enhanced phase change material, temperature of the water at the outlet, and heat transfer capability decreased in the solar water heater. The following paragraphs will describe the process of producing the nanoparticles and the final nano-enhanced phase change material. The copper oxide nanoparticles were synthesized through electrochemical discharge machining using a copper wire as a cathode and a copper sheet as an anode. Both electrodes were dipped into a 0.5 M potassium hydroxide solution that acted as an electrolyte. The electrochemical discharge machining technique works on the principle of erosive action under which a high potential difference is maintained between the electrodes. Because of this, the heat is liberated, causing the material to melt and evaporate from both electrodes. The arc discharge takes place at the cathode surface by supplying a direct current power supply and maintaining a threshold voltage of 85 V across the electrodes. The molten copper is deposited into the electrolytic solution. The nanoparticle-mixed electrolyte is centrifuged at 10,000 rpm until the droplets of the molten copper metal settle down at the bottom of the test tube in the form of black precipitates because of the sudden cooling. The precipitates are segregated, washed, and dehydrated in an oven at 80 °C for 24 h. The effect of electrolyte temperature has been investigated by using an aqueous potassium hydroxide solution, which is heated to 40 °C with a magnetic stirrer. To ensure optimum mass output rate and to prevent the agglomeration of the nanoparticles, a magnetic stirrer was rotated at 220 rpm. The copper oxide–phase change material composite was synthesized by using the gravity die casting process. In the hot-chamber gravity die casting, the base paraffin wax was utilized and allowed to melt. As the paraffin wax melted completely, the copper oxide nanoparticles were introduced into the cavity chamber through the provided passage and finally rotated so that they were mixed properly with molten paraffin wax with the help of a stirrer. After the mixing, the copper oxide–phase change material was drained out from the crucible and poured around the periphery of a copper pipe.

### 2.5. Vacuum Impregnation

**Figure 6:**
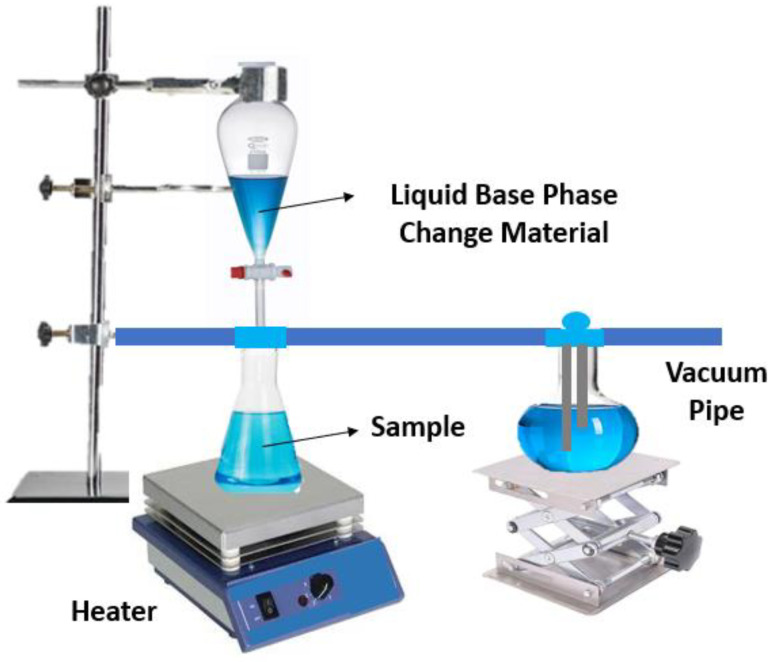
Vacuum impregnation set-up.

The vacuum impregnation method is used to improve the incorporation of nanoparticles into the base phase change material. It has beneficial features of enhancing the long-term phase stability of the nano-enhanced phase change materials and preventing the molten base phase change materials from leaking at high pressure. Additionally, vacuum impregnation equipment has been developed to produce high amounts of shape-stabilized phase change materials without the need for extra filtering and crushing processes. The authors Kim et al. [38] followed a two-step procedure to synthesize a nano-enhanced phase change material composed of hexadecane with the addition of exfoliated graphene nanoplatelet nanoparticles by the vacuum impregnation technique. Before the nanocomposite preparation, the exfoliated graphene nanoplatelets were prepared by applying cost-effective exfoliation to commercially available graphite. To produce the nano-enhanced phase change material, the exfoliated graphene nanoplatelets were placed inside a filtering flask that was then vacuum-pressurized for one hour before the mixing process with liquid hexadecane. After that, the mixture was filtered and dried in a vacuum drier at 80 °C for one day. Figure 6 schematically illustrates the vacuum impregnation equipment and procedure.

## 3. Phase Change Material Nanofluid Thermophysical Properties

### 3.1. Thermal Conductivity

**Figure 7:**
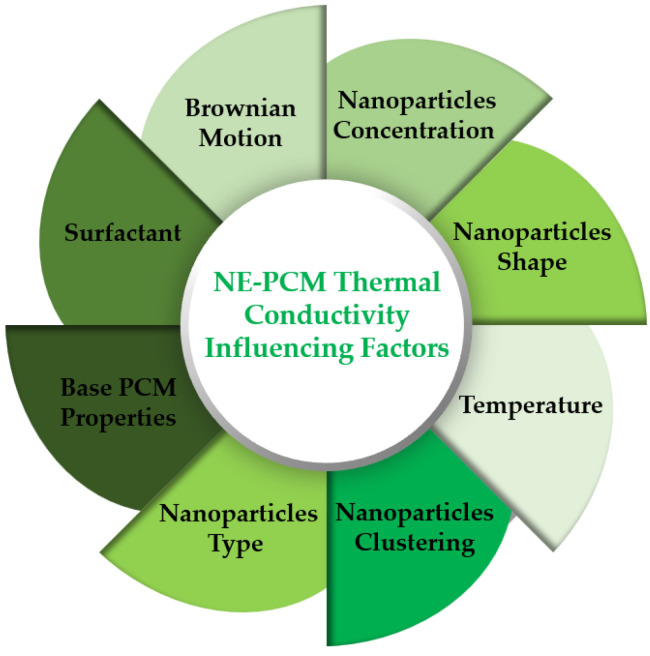
Main thermal conductivity influencing factors of the nano-enhanced phase change materials.

A major part of the available scientific articles is focused on the thermal conductivity increase as a direct consequence of the addition of nanoparticles to the base phase change materials. One exception is, for example, the experimental work performed by the authors Colla et al. [39], in which the addition of alumina nanoparticles to a base of paraffin provoked up to 8% lower thermal conductivity when compared to that of the paraffin itself. However, the authors did not provide a clear interpretation of such a fact. Broadly speaking, because of the superior thermal conductivity of nano-enhanced phase change materials, it is expected to decrease the heating, melting, and solidification durations. Also, the reported thermal conductivity enhancements were up to 100% or even more. This was the case for, e.g., lauric acid base phase change material with the inclusion of graphene nanoplatelets [40], carbon phase change materials [41], or magnesium chloride hexahydrate phase change materials [42]. Usually, it can be concluded that an increment in the concentration of the nanoparticles in the phase change materials will increase the thermal conductivity. Nonetheless, there is a limiting concentration of added nanomaterials in the base phase change material, given that the excessive values of concentration beyond this limit induce the avoidable agglomeration of the nanostructures. Such an agglomeration effect led to enhanced thermal conductivity in some published cases and reduced thermal conductivity in others. This dual impact on the thermal conductivity of the nano-enhanced phase change materials could be misleading and lead to inconsistent conclusions. It is difficult to accurately predict when the agglomeration of nanoparticles will happen, given that this effect depends on several factors like the morphology and concentration of the nanoparticles, and type, phase time, and temperature of the base phase change materials. The authors Harish et al. [40] observed the thermal conductivity of lauric acid phase change material augmented with increasing concentrations of graphene nanoplatelets. The researchers found a drastic increase of about 230% for the nano-enhanced phase change material at 1% vol. of graphene nanoplatelets. On the other hand, the authors Sharma et al. [43] fabricated a nano-enhanced phase change material of palmitic acid with the addition of titanium oxide nanoparticles and stated that the increasing concentration of the nanoparticles caused the thermal conductivity of the nano-enhanced phase change material to increase. Also, the authors reported that at 5% wt. of titanium oxide nanoparticles, the maximum thermal conductivity increment of nearly 80% was reached. Nonetheless, at the same concentration of nanoparticles, the authors reported a 15.5% decrease in the latent heat of fusion compared to that of the phase change material itself. Additionally, the researchers Lin et al. [44] examined the thermophysical characteristics of nano-enhanced phase change materials composed of paraffin wax and copper nanoparticles. The research team found that the resultant thermal conductivity increased with increasing concentrations of nanoparticles. The authors also observed that increasing the concentration of the nanoparticles decreased the latent heat. The peak thermal conductivity enhancement of nearly 46% was obtained at 2% wt. of nanoparticles. Furthermore, the researchers Wang et al. [31] fabricated a phase change material of OP10E and water emulsion and graphite nanoparticles. The authors argued that the supercooling effect of the emulsion could be prevented for a concentration greater than 2% wt. of graphite nanoparticles. The research team also confirmed that the incorporation of the graphite nanoparticles into the OP10E/water emulsion did not affect the latent heat capacity of the system. It was also observed that the inclusion of 2% wt. of nanoparticles led to a thermal conductivity enhancement of nearly 89%. Additionally, the researchers Sami and Etesami [45] investigated the thermophysical properties of a nano-enhanced phase change material composed of paraffin and titanium oxide nanoparticles and verified a thermal conductivity increase of nearly 48% with 2% wt. of nanoparticles. Also, the authors observed no considerable alterations in the temperature of melting and latent heat capacity after pre-defined thermal cycling. Moreover, the authors Barreneche et al. [46] argued that the shape and size of carbon black nanoparticles, multi-walled carbon nanotubes, and graphene oxide nanosheets considerably influenced the thermal conductivity of nano-enhanced phase change materials. They determined thermal conductivity increases of 8%, 14%, and 7% at 0.1% wt. of carbon black/water, multi-walled carbon nanotubes/water, and graphene oxide/water, respectively. Moreover, the researchers Harikrishnan et al. [47] investigated the thermophysical properties of myristic acid with the incorporation of silica nanospheres. The highest reported enhancement in the thermal conductivity of the nano-enhanced phase change material was around 87% and it was attained at 1.0% wt. of incorporated silica. Furthermore, the researchers Salyan and Suresh [34] evaluated the thermophysical properties of D-Mannitol with copper oxide nanoparticles. The inclusion of 0.5% wt. of the nanoparticles to the phase change material led to a 25% enhancement in thermal conductivity. The incorporation of the nanoparticles induced a minor decrease in the latent heat of the D-Mannitol base phase change material. The researchers Mayilvelnathan and Arasu [48] found that the thermal conductivity of erythritol, measured with the laser flash method, was enhanced with the incorporation of graphene nanoparticles between 0.1% wt. and 1% wt., before and after thermal cycling periods. The thermal conductivity of erythritol was 0.733 W/mK, whereas the thermal conductivity of the final nano-enhanced phase change material was increased to 1.074 W/mK, 1.095 W/Mk, and 1.122 W/mK at 0.1% wt., 0.5% wt., and 1% wt., respectively. After the thermal cycling, the thermal conductivity was 0.692 W/mK for erythritol, and 0.899 W/mK, 0.921 W/mK, and 1.020 W/mK for erythritol with graphene nanoparticles at 0.1% wt., 0.5% wt., and 1% wt., respectively. Additionally, the authors Putra et al. [49] studied the thermophysical characteristics of nano-enhanced phase change materials composed of Rubitherm™ paraffin wax, RT22 HC, with the inclusion of graphene nanoplatelets. The thermal conductivity of the paraffin wax was increased by nearly 90% with the addition of 0.3% wt. of graphene nanoplatelets. In addition, the researchers Yadav et al. [42] synthesized nano-enhanced phase change materials of magnesium chloride hexahydrate and nano-graphite. The nano-enhanced phase change material at 0.5% wt. of nano-graphite particles exhibited a substantial thermal conductivity enhancement of 308%. It also showed increases in the melting and solidification rates of 22% and 75%, respectively, compared to those achieved with the base phase change material itself. Additionally, the authors Praveen and Suresh [35] performed experimental research studies on the heat transfer behavior of neopentyl glycol enhanced with copper oxide nanoparticles. The researchers conducted thermal storage and release experiments and determined the thermal conductivity of the nano-enhanced phase change material. At 3% wt. of copper oxide nanoparticles, the storage time was decreased by nearly 34%, and at the same concentration, the thermal conductivity of the base phase change material experienced a 4-fold increase. Furthermore, the authors Nourani et al. [29] enhanced paraffin with different weight concentrations of alumina nanoparticles. The researchers claimed that the correlation between the effective thermal conductivity and increasing concentration of alumina nanoparticles was nonlinear. The increment in the effective thermal conductivity was 31% in the solid phase and 13% in the liquid phase for the nano-enhanced phase change material incorporating 10% of alumina. At the same weight fraction of nanoparticles, the authors also reported a 27% decrease in the heating and melting times in the base phase change material. Finally, the researchers Wang et al. [50] measured the thermal conductivity of paraffin and titanium oxide nanoparticles at distinct concentrations and temperatures. The research team confirmed that the thermal conductivity decreased with increasing temperatures up to 60 °C because of the randomly wised motion of the nanoparticles in the base fluid. The authors also observed that the thermal conductivity increased with increasing titanium oxide concentrations and estimated the theoretical thermal conductivity of the nano-enhanced phase change materials given by Equation (1):

k_NEPCM_ = Φ_v_ × k_p_ + (1 − Φ_v_) × k_f_ (1)

where Φ_v_ is the volumetric concentration of the included nanoparticles, and k_p_, k_f_, and k_NEPCM_ are the thermal conductivity of the nanoparticles, phase change material, and nano-enhanced phase change material, respectively. In addition, the volumetric concentration and mass fraction Φ_w_ can be calculated by Equation (2):

Φ_v_ = Φ_w_ × (ρ_NEPCM_/ρ_p_) (2)

where ρ_NEPCM_ is the nano-enhanced phase change material density and ρ_p_ is the nanoparticle density. The estimated thermal conductivity was higher than the experimentally measured one for high nanoparticle concentrations. Additionally, the authors Wang et al. [50] noted that Equation (1) can accurately estimate the thermal conductivity at nanoparticle concentrations less than 3% wt. At higher concentration levels, Equation (1) overestimates the resulting thermal conductivity. At higher concentrations, nanoparticle clustering is facilitated because of the attractive and repulsive forces between the nanoparticles themselves. The clustering often increases the thermal resistance, provoking a negative impact on the thermal conductivity improvement. Figure 7 summarizes the main influencing parameters of the thermal conductivity of the nano-enhanced phase change materials.

### 3.2. Specific Heat

It should be emphasized that only a few more studies addressed the results for the specific heat of the nano-enhanced phase change materials. Moreover, the authors Chieruzzi et al. [32], as already mentioned, fabricated a 60% wt. NaNO_3_–40% wt. KNO_3_ nitrate salt eutectic mixture with the addition of alumina, silica, and alumina/silica nanoparticles at a concentration of 1% wt. The best specific heat results were obtained with the alumina/silica nanoparticles, for which an increase of around 52% was verified for the solid phase and an increase of around 19% was observed for the liquid phase. Also, the amount of stored heat was enhanced by 13.5% compared to that achieved with the phase change material alone. Finally, the researchers Liu and Yang [25] improved the specific heat of an inorganic hydrate salt with titanium oxide–P25 nanoparticles. At 0.3% wt. of nanoparticles, the specific heat was increased by up to nearly 84% in the solid phase and around 15% in the liquid phase. 

### 3.3. Latent Heat

**Figure 8:**
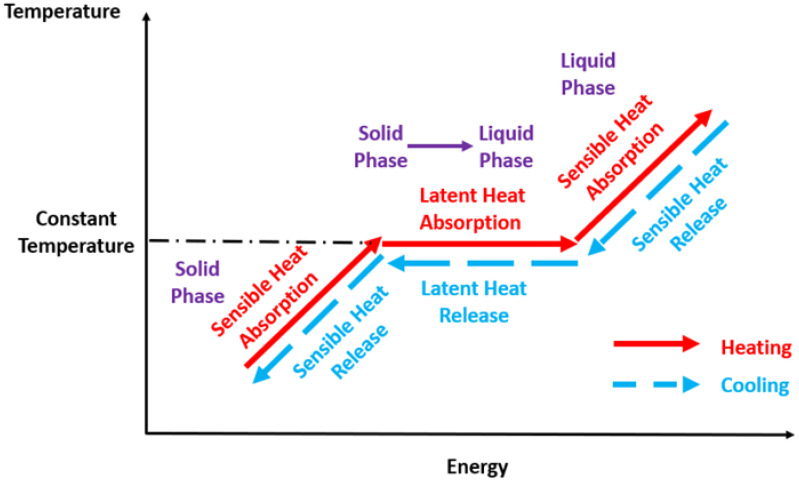
Plot depicting the optimal curves of the temperature variation of the phase change materials during heating and cooling.

When heating or cooling a material, two distinct types of energy under the form of heat should be considered: latent heat and sensible heat. Latent heat does not entail a temperature alteration during heat absorption or dissipation and is normally employed to overcome the intermolecular forces of a material during its melting. The heat is released during the solidification process, and consequently, the molecules of the material can be orderly rearranged. Oppositely, sensible heat alters the temperature of the material. Also, latent heat can be divided into latent heat of fusion of solid-to-liquid or liquid-to-solid phase changes and latent heat of vaporization of liquid-to-gas, or, alternatively, gas-to-liquid phase changes. The research community has been fundamentally concerned with the latent heat of fusion, given that the application of the phase change materials is limited to the solid and liquid states. One of the most prominent thermophysical properties of phase change materials is their inherent latent heat capacity. Hence, any inclusion of nanostructures should not deteriorate the latent heat of the base phase change material. Concerning the latent heat behavior of the nano-enhanced phase change materials, the researchers usually explained their latent heat alterations based on the different interaction between the molecules of the phase change material and nanoparticles and on the concentration of the latter, given that an excessive fraction of nanoparticles may induce a latent heat deterioration. The concentration and morphology of the nanoparticles and their intermolecular interaction with the phase change materials are factors to consider in the latent heat behavior of nano-enhanced phase change materials. Additionally, an increased surface-to-volume ratio of the nanoparticles and the addition of surfactants promote the increase in the latent heat of the phase change material. Apart from these characteristics, it should be mentioned that some researchers observed that the nanoparticles decreased the latent heat of the phase change material [6,12,28,51]. Such a reduction trend was observed even at low concentrations of nanoparticles up to 1% wt. Some of the decrement can be taken as negligible, whereas others can be up to 5%. For instance, the latent heat of fusion of paraffin decreased by only 0.9% when multi-walled carbon nanotubes up to 2.0 wt.% were added [52]. Also, as it is declared in a major part of the published works, the incorporation of nanoparticles provoked a reduction in the latent heat, except in the works conducted by the authors Shaikh et al. [53] and Mohamed et al. [54], who observed increases of approximately 13% and 2%, respectively. Figure 8 presents a plot with the optimal curves of the temperature variation in the phase change materials during the heating and cooling processes.

Moreover, the researchers Warzoha et al. [28] reported that the latent heat of organic paraffin decreased with increasing amounts of herringbone graphite nanofibers. The latent heat of fusion of the pure paraffin was 271.6 J/g, whereas the latent heat of fusion of the nano-enhanced phase change material at 11 vol% of graphite nanofibers was 242.7 J/g. The researchers Colla et al. [39] incorporated carbon black and alumina nanoparticles in the RT20 and RT25 paraffin waxes from *Rubitherm*^®^. The alumina nanoparticles incorporated in the RT20 paraffin wax improved its latent heat by nearly 11%, whereas the carbon black nanoparticles enhanced the latent heat of the same paraffin wax by only around 3%. Moreover, the inclusion of carbon black nanoparticles in the RT25 paraffin wax caused a nearly 12% reduction in the latent heat. Additionally, the authors Ebadi et al. [33] incorporated copper oxide nanoparticles into coconut oil to synthesize a nano-enhanced phase change material. At 1% wt., the enhanced material exhibited a nearly 8% decrease in the latent heat of fusion as compared to that of the phase change material alone. Also, the authors Wang et al. [50] found that the addition of 0.7% wt. of titanium oxide nanoparticles to the base paraffin wax produced an increment in the latent heat. Nonetheless, a weight concentration greater than the mentioned one caused the latent heat to deteriorate. The researchers Harikrishnan et al. [47] reported that the latent heat during the melting and solidification of myristic acid with 1% wt. of silica nanoparticles was reduced by around 1% and 0.6%, respectively, compared to myristic acid itself. Additionally, the researchers Lin and Al-Kayiem [44] found a reduction of around 15% in the latent heat of fusion at 2% wt. of copper nanoparticles incorporated in paraffin wax. The authors also verified that the latent heat of solidification decreased by nearly 13% at the weight fraction. Moreover, the researchers Shaikh et al. [53] found that concentrations up to 1% vol. of single-walled carbon nanotubes, multi-walled carbon nanotubes, and carbon nanofiller increased the latent heat of fusion of the paraffin wax base phase change material. The reported enhancements were nearly 13%, 10%, and 7% for the single-walled nanotubes, multi-walled nanotubes, and nanofiller, respectively. This approach was also adopted by Israelachvili [55], who addressed the enthalpy alteration caused by the interaction between the surface of the carbon nanotubes and the base phase change material. Usually, if the different molecules at stake are not likely to interact, the short-range forces where the molecule centers are separated by up to 3 Å are repulsive forces, and the long-range or van der Walls forces are attractive forces. The potential energy of the van der Walls forces that is in ionic and in covalent bonds is recognized by latent energy. In the solid-to-liquid phase change transition, the energy under the form of heat that is absorbed is employed to overcome the weak intermolecular attractions. It should be noted that a high enough addition of nanoparticles in the base phase material may change the long-range forces. Hence, a latent heat enhancement is expected when the interaction potential between the included nanostructures (e.g., carbon nanotubes) and the base paraffin wax is greater than the potential between the molecules of the paraffin themselves. Additionally, the authors Wang et al. [52] evaluated the effects on the latent heat of multi-walled carbon nanotube dispersions in water and concluded that the latent heat inconsistency increased with increasing concentrations of multi-walled carbon nanotubes. Similar characteristics were also observed by the researchers Li et al. [56], who dispersed multi-walled carbon nanotubes in stearic acid. The main cause for these characteristics may be the fact that the multi-walled carbon nanotubes employed by Wang et al. [52] were large, 30 nm, and the surface area was small, and hence, the latent heat enhancement was less considerable in comparison to those reported by, for example, the researchers Shaikh et al. [53]. Hence, it can be said that the variation in diameter of the carbon nanotubes impacts the latent heat of the phase change material. In the study carried out by the authors Zeng et al. [57], it was found that the latent heat would drastically decrease as compared to that of palmitic acid as the concentration of multi-walled carbon nanotubes was enhanced. When the concentration of the carbon nanotubes reached the limiting value, the latent heat increased unexpectedly, achieving a peak value. With a further increase in the content of nanotubes, the latent heat decreased linearly with increasing concentrations of carbon nanotubes. According to the authors Zeng et al. [57], low fractions of multi-walled carbon nanotubes will disperse more uniformly in the base palmitic acid, making the carbon nanotubes absorb more palmitic acid in the process. This will lead to more palmitic acid molecules losing their phase change capability. This is why the latent heat of the enhanced materials would substantially decrease at low carbon nanotube concentrations. Nevertheless, when the fraction of carbon nanotubes is increased, it will cause the clustering of the nanotubes. This will lead the multi-walled carbon nanotubes to absorb less palmitic acid. Consequently, the latent heat will be increased to a maximum. Further concentration increases in the multi-walled carbon nanotubes would not affect their agglomeration, so the latent heat would decrease. Hence, it may be assumed that the latent heat of nano-enhanced phase change materials will vary with the concentration of carbon nanotubes. Additionally, the authors Meng et al. [58] synthesized fatty acids with the addition of carbon nanotubes. The employed fatty acids were a combined formulation of lauric acid, capric acid, and palmitic acid, and the authors found that the phase change energy was reduced for higher fractions of carbon nanotubes. The porous structure of the carbon nanotubes decreased the crystal formation of the fatty acids during freezing. Such an effect was reduced when the weight fraction of the fatty acids was augmented. Moreover, the authors do not explain in a clear way how the surface modification of nanoparticles could impact the latent heat capacity of the base phase change material. Already reported in the literature was the deterioration of the latent heat of the nano-enhanced phase change materials having metal and metal oxide nanoparticles like silver, copper, aluminum, copper oxide, and titanium oxide dispersed in diverse base phase change materials. Additionally, the researchers Wu et al. [59] added copper nanoparticles to paraffin and found maximum decreases of approximately 11% and 12% in the latent heat of the melting and freezing processes, respectively. The authors reported that the latent heat dropped with increasing concentrations of copper nanoparticles. Moreover, the authors Parameswaran et al. [60] examined surface-functionalized silver nanoparticles dispersed in organic ester. In the freezing and melting cycles, the decrease in the latent heat varied from around 1.8% to 7.9% and from nearly 1.2% to 8.9% with 0.1% wt. and 5% wt. of nanoparticles, respectively. The authors Ho and Gao [61] studied the thermophysical properties of paraffin (C_18_H_38_, n-octadecane) emulsion with the addition of alumina nanoparticles. It was found that the latent heat of the base paraffin was reduced with increasing concentrations of alumina nanoparticles. The mentioned reduction was nearly 13% with 10% wt. of alumina nanoparticles. Moreover, the researchers Yang et al. [62] dispersed Si_3_N_4_ nanoparticles in paraffin and found that the latent heat was increased with 1% wt. of nanoparticles. The phenomenon could be explained based on the principles of thermodynamics by assuming that the mixing of the silicon nitride in the base paraffin is a constant pressure process and that there may be some compatibility between the nanoparticles and the paraffin. Considering the equation of thermodynamics, the principle of the system entropy shows that when adding 1% wt. of silicon nitride nanoparticles to the base paraffin, the mixture tended to be under a chaotic state. Assuming the silicon nitride and paraffin as a system, the entropy increases and DS is greater than zero. Also, the increased volume DV could be negligible for the solid–solid and solid–fluid nanocomposites. Hence, the authors concluded that TDS > pDV for DU > 0. Consequently, the enthalpy of the system increased, and the latent heat of the nano-enhanced material at 1% wt. of silicon nitride was greater than that of the pure paraffin. Further raising the concentration of the silicon nitride nanoparticles, the increase in the pDV term would be greater than the increase in the TDS term. Hence, the enthalpy and the latent heat decreased with increasing silicon nitride concentration. Finally, it should be stated that when dispersing the carbon nanotubes, carbon nanofibers, metal, or metal oxide nanoparticles in a base phase change material, it was verified that the calculated latent heat values were always higher than the experimentally obtained ones. Also, such inconsistencies were found to increase with increasing concentrations of nanoparticles. 

### 3.4. Viscosity

When the viscosity is high, extra pumping power is required to set the fluid in motion. Nonetheless, the use of a phase change material, even in the liquid state, does not require it to flow continuously. The phase change material is stored in a specially conceived container during the melting or solidification. The viscosity of the nano-enhanced phase change materials depends fundamentally on the concentration of the nanoparticles and working temperature. Also, the viscosity of the liquid phase change material impacts the stability of the nanoparticles in the fluid. According to Stokes’ law, the sedimentation rate of the nanoparticles diminishes when a viscous fluid is employed. Thus, the nanoparticles exhibit a greater tendency to move downward because of the gravitational force when the fluid possesses low viscosity values. The incorporation of nanoparticles in the liquid phase change material will enhance its viscosity and promote the disruption of the liquid flow. The viscosity affects the natural convection, and this plays a significant role in the melting process of a phase change material [63]. The viscosity is frequently estimated by the Brinkman correlation for suspensions [64]. A high viscosity may also hinder the sonication procedure to de-agglomerate the nanoparticles. For example, the authors He et al. [65] observed that the viscosity of titanium oxide nanoparticles dispersed in an aqueous solution of barium chloride increased with increasing volume concentrations of the nanoparticles. The investigation team explained the observed trend with the fact that as the concentration of nanoparticles was raised, the distance between them decreased. Under such conditions, the frictional force between the nanoparticles and between the nanoparticles and the molecules of the water in the aqueous solutions was swiftly increased. This was manifested by the macroscopic increase in the viscosity. The authors Ho and Gao [61] found that the viscosity of a paraffin emulsion had a nonlinear trend with the addition of alumina nanoparticles at diverse concentrations. Additionally, the viscosity decreased with increasing temperature. Also, the increase in the viscosity was much more intense than the increase in the thermal conductivity under the same operating conditions. A similar trend was observed by the researchers Jesumathy et al. [66] using water with the addition of copper oxide nanoparticles. The researchers found that the viscosity increased dramatically when the weight fraction of the nanoparticles was greater than 10% wt. In addition, the authors Bahiraei et al. [67] measured the viscosity of paraffin, with a melting point of 60 °C, with the inclusion of carbon nanofibers, graphene nanoparticles, and graphite nanopowder. The increase in the concentration of the incorporated nanostructures increased the dynamic viscosity, but as the temperature was augmented from 60 °C to 90 °C, the dynamic viscosity was slightly decreased because of the enhancement in the kinetic energy of the nanoparticles. Moreover, the researchers Motahar et al. [30] analyzed the viscosity of n-octadecane containing mesoporous silica. The experimental results indicated that with a lower concentration of nanoparticles, the behavior of the enhanced material was Newtonian. Nonetheless, with concentrations greater than 3% wt., the behavior was non-Newtonian. Also, the researchers Daneshazarian et al. [68] investigated the heat transfer behavior of nano-enhanced phase change material octadecane-exfoliated graphene nanoplatelets up to a concentration of 2 wt.%, with 0.5 wt.% being the ideal concentration value. Incorporating more nanoparticles could increase the viscosity, reducing the overall effect of the conductivity enhancement. The research team concluded that due to the complexity of the thermal behavior and multiple influencing factors in the heat transfer of nano-enhanced phase change materials, thermal conductivity should not be the only criterion considered.

### 3.5. Density

The density of nano-enhanced phase change materials is expected to grow with increasing concentrations of nanoparticles. Given that the phase change material storage system possesses a limited volume value, the phase change material that has the higher density is often the most adequate option [69], enabling it to provide an augmented mass transfer during the phase change process [70]. However, there are only a moderate number of experimental works that emphasize the density characterization of such materials. Furthermore, the authors Warzoha et al. [28] stated that the density of paraffin with the inclusion of graphite nanoparticles at 8 wt.% increased by up to nearly 1.5% compared to the density of the paraffin itself. It was also reported that the density of the paraffin with the incorporation of graphene, multi-walled carbon nanotubes, and aluminum and titanium oxide nanostructures was higher than that of the base paraffin at 20% vol. Moreover, the researchers Owolabi et al. [71] measured the impact of the addition of nanoparticles of aluminum, zinc, iron, and copper having concentrations of 0.5% wt., 1% wt., and 1.5% wt. on the density of paraffin-based nanocomposites. The incorporation of the nanoparticles demonstrated all the same behavior, increasing the final density at 1 wt.% and slightly decreasing it at 1.5 wt.% as compared to the density at 1 wt.%. Also, the researchers Abdelrazik et al. [72] evaluated the density of nano-enhanced phase change materials composed of paraffin and graphene nanoparticles with initial densities of 800 kg/m^3^ and 2400 kg/m^3^, respectively. The concentration of the graphene nanoparticles was between 1% wt. and 20% wt., and the results revealed that the density of the final material increased from 800 kg/m^3^ to 923 kg/m^3^.

### 3.6. Sub-Cooling and Phase Change Temperature and Time

Sometimes, the phase change materials will start to solidify at a temperature substantially lower than its point of melting, and this is caused by the subcooling or supercooling effect. The phase change material will not release the stored latent heat at the melting point because of the subcooling. The subcooling induces the material to start crystallization considerably below the phase change temperature. The crystallization process commences with the nucleation stage, which can be divided into homogeneous and heterogeneous nucleation. Homogenous nucleation is initiated by the phase change material itself because of the sufficiently low temperature, or, for the secondary nucleation, the solid phase change material, which is added to the subcooled phase change material. Heterogeneous nucleation commences not within the phase change material itself but with the inclusion of special additives added to the material. In the nucleation process, the free energy variations are related to the nuclei formation. Also, there is a critical free energy value when stable nuclei of critical size are formed. Regarding the nano-enhanced phase change materials, some authors described the added nanoparticles as nucleation agents that provoke a decrease in the melting and solidification time. The nanoparticles could act as heterogeneous nucleation agents in the nano-enhanced phase change materials. In a study using n-hexadecane as phase change material and multi-walled carbon nanotubes, the authors Zhang et al. [73] observed a decrease in the subcooling of the phase change material, given that the carbon nanotubes provided proper-sized stable nuclei. The nuclei facilitated heterogeneous nucleation and, consequently, strongly promoted the crystallization process. Also, the authors Wu et al. [74] claimed that in the freezing of water with a dispersion of alumina nanoparticles, the enhanced material exhibited a lower supercooling degree than the water itself since the nanoparticles acted as nucleating agents. For this to happen, the nanoparticles should have a similar structure to that of the base phase change material. In addition, the initial freezing point depends mainly on the size of the nanoparticles. Moreover, subcooling negatively influences the effectiveness of the phase change materials in the thermal energy storage field [75]. The authors Kumar and Kalaiselvam [76] referred to it as the difference between the peak temperature at melting and the peak temperature at solidification. The sub-cooling effect is a more prominent factor in the cooling process rather than in the heating process [77]. In cases where the subcooling degree is significatively high, it is harder for the phase change material to release the stored heat even at its solidification point. The dissipation of the latent heat only commences at a temperature value less than the solidification temperature. The authors Kumar and Kalaiselvam [76] observed the formation of ice crystals in a nano-enhanced phase change material during its cooling. The authors interpreted this formation as based on the nucleating action of the dispersed nanoparticles in the base phase change material. In addition, a thickening agent like the 2-hydroxypropyl ether cellulose incorporated in eutectic phase change materials also facilitates the crystallization in the phase change material [78]. There are also reports revealing a shorter duration is required for the complete melting and solidification processes of nano-enhanced phase change materials. As mentioned earlier, these materials have a higher thermal conductivity compared to traditional phase change materials. Improved thermal conductivity aids in the absorption/release of heat processes. Additionally, the researchers Harikrishnan et al. [47] implied that reductions of nearly 30.7% and 32.3% were attained for melting and solidification times, respectively, at 1% wt. of silica nanoparticles in myristic acid compared to myristic acid alone. Similarly, the researchers Warzoha and Fleischer [79] compared paraffin wax with diverse enhanced paraffin waxes with the addition of graphene, aluminum, and titanium oxide nanoparticles and multi-walled carbon nanotubes. The authors observed no peculiar trend regarding the effect of the nanoparticles on the solidification and melting points, only reporting minor variations. For example, the researchers Wang et al. [52] found that the melting temperature of paraffin decreased from 53 °C to 52 °C at 2% wt. of multi-walled carbon nanotubes. Also, the researchers Mohamed et al. [54] used paraffin as a phase change material and observed a slightly lower melting temperature of 51.7 °C at the same weight fraction, but this time with the incorporation of α-alumina nanoparticles. Moreover, the authors Lin and Al-Kayiem [44] found a decrease of 4.3% in the melting temperature with 2% wt. of copper nanoparticles dispersed in paraffin wax, whilst the solidification point increased by 2.3%. The greater determined variations might be caused by the distinct phase change temperatures of the paraffin wax employed in the different studies. Also, the researchers Harikrishnan et al. [47] reported that the melting and solidification temperatures increased by nearly 1.1% and 1.3%, respectively, when 1% wt. of silica nanoparticles was added to the myristic acid. A slightly higher decrease in the melting and solidifying temperatures was verified by the authors Rufuss et al. [80], who investigated paraffin with the incorporation of graphene oxide, copper oxide, and titanium oxide nanostructures. The decrement range was from around 5% to 9%. Generally, it appears that the changes in the phase change temperatures depend mainly on the nature of the phase change material, and the type and fraction of the nanoparticles. Additionally, the authors Wu et al. [74] found that the size of the used alumina nanoparticles was 20 nm, which was closer to the water particle size. Consequently, in this specific case, the surface free energy between the nanoparticles and the water was low, which led to improved wetting, resulting in a subcooling degree decrease. Similar effects were found for titanium oxide nanoparticles as nucleating agents in the phase change material by the researchers Liu et al. [81] and He et al. [65]. Furthermore, the authors He et al. [65] reported that the size of the employed titanium oxide nanoparticles was approximately 20 nm, which was close to the particles of the BaCl_2_/water suspension. Under these conditions, the surface free energy between the crystal nucleus and the nanoparticles was very low and the wetting/contacting between the nanoparticles and the phase change material was improved. The contact angle was close to zero degrees and the subcooling of the nano-enhanced materials also tended to be zero. Additionally, the authors Hu et al. [82] carried out experimental work on the phase change behavior of sodium acetate trihydrate, which possessed superior thermal conductivity and storage density, but a high subcooling degree and phase segregation. Thus, it was recommended to use nucleating agents to prevent subcooling. In this case, the aluminum nitride nanoparticles were chosen to be the nucleating agents and the authors observed that at 5% wt., there was no tendency for subcooling. Also, the average crystal size of the nano-enhanced phase change material was much smaller than that of the base phase change material itself, which promoted the prevention of phase segregation. In addition, the researchers Parameswaran et al. [60] studied the thermal performance of organic ester with the inclusion of silver nanoparticles. As verified by the FTIR technique, the molecules of the silver nanoparticles had no interaction with the molecules of the base phase change material. Thus, it is reasonable to assume that the enhanced thermal conductivity and heat transfer rate of the molecules of the silver nanoparticles contributed to the supersaturation condition to be initiated in the nano-enhanced phase change material. Such a fact promotes the establishment of the chemical potential and thermodynamic equilibrium between the solid and liquid phases. Hence, the free energy of the molecules was reduced to enable the formation of nuclei having a radius greater than the critical radius, and this allowed the growth of the nuclei during freezing. The additional energy needed for the formation and growth of stable nucleus crystal in freezing was increased by the molecules of the silver nanoparticles. These promoted the formation of an orderly structuring of the phase change materials molecules and, consequently, allowed the nano-enhanced phase change material to crystallize at a faster rate. Also, the activation energy released by the stable nuclei at the nucleation and crystal growth stages could be attributed to the latent heat of the enhanced material. This is an exothermic reaction from the standpoint of the molecules of the material. The nucleation effect of graphite nanoparticles dispersed in polyethylene glycol was examined by the researchers Zhang et al. [73]. With a large aspect ratio, the nanoparticles gave an extended surface for the crystallization process of the base polyethylene glycol. Hence, the nucleation could be facilitated by increasing the concentration of nanoparticles and reducing the subcooling degree. Moreover, the authors Jesumathy et al. [66] exposed that the heat storage features might play a relevant role in diminishing the subcooling effects. Additionally, the researchers Wang et al. [27] reported that the phase change temperature of paraffin shifted to a lower value because of the inclusion of alumina in the paraffin wax, except at a concentration of 1% wt. of nanoparticles. Table 1 summarizes some of the published experimental works on the nano-enhanced phase change materials’ thermophysical properties.

### 3.7. Stability of the Nano-Enhanced Phase Change Materials in Acids and Salts

The thermal stability of the nano-enhanced phase change materials having acids and inorganic salts as base phase change materials can be evaluated by techniques such as differential scanning calorimetry and thermogravimetric analysis. The stability over time of the nano-enhanced phase change materials is normally evaluated by visual inspection of the nanostructures that have been doped into the molten phase for extended periods. First considering acids, for example, fatty acids are organic phase change materials, which have attracted more attention from researchers because of their inherent increased latent heat, suitable transformation temperature, low degree of supercooling, minimal volume changes, and enhanced thermal stability. The transformation temperature of fatty acids commonly used in phase change thermal energy storage material ranges from 30.1 to 70.7 °C, and their phase change latent heat ranges from nearly 149 to 223 J/g. The thermal conductivity of the fatty acids is relatively poor and the most widely used method for improving the thermal conductivity of fatty acid phase change materials is the addition of high thermally conductive nanostructures. These usually include metal nanoparticles, metal oxide nanoparticles, and carbon materials like carbon nanotubes and expanded graphite. The expanded graphite is widely employed as a form-stable matrix due to its low density, high thermal conductivity, and porous hierarchical structure, preventing phase change material leakage and considerably enhancing the effective thermal conductivity. Moreover, the authors Zhou et al. [93] dispersed expanded graphite in myristic acid and evaluated the thermal stability of the nano-enhanced phase change material through thermogravimetric analysis. The myristic-acid-expanded graphite nano-enhanced phase change material was heated from ambient temperature to 80 °C and it was maintained for one hour at that temperature. The results showed that the myristic acid started to lose weight at around 120 °C. The weight loss reached its peak at approximately 211 °C, and the acid was almost totally volatilized at around 244 °C. Such results showed that for operating temperatures lower than 120 °C, even if the melting temperature of the myristic acid is surpassed, the developed nano-enhanced phase change material will not lose myristic acid. Hence, the authors concluded that the myristic-acid-expanded graphite-enhanced material had good thermal stability in heat transfer applications at less than 100 °C. Moreover, the thermal cycle reliability of the phase change materials’ evaluation determines the attenuation of the heat storage capability after repeated heat storage/releasing cycles. The thermal cycle acceleration experiments infer the thermal cycle reliability of the phase change materials, concerning the phase transition temperature before and after one complete thermal cycle and the phase change latent heat. For such an evaluation, the research team determined the differential scanning calorimetry curves and thermal performance defined by the melting and freezing temperatures, and the latent heat of melting freezing of the nano-enhanced phase change material after 50, 100, and 200 thermal cycles. The authors reported that the differential scanning calorimetry curves of the material before and after a thermal cycle were very close. Also, after 50, 100, and 200 thermal cycles, the phase change temperatures changed to 0.3 °C, 0.1 °C, and 0.5 °C, respectively, and the latent heat changed to 0.1%, 0.9%, and 4.5%, respectively. This outcome indicated that the myristic-acid-expanded graphite phase change materials possessed good thermal cycle reliability for long-term operation. Furthermore, the researchers Martín et al. [8] synthesized a nano-enhanced phase change material composed of a capric acid and myristic acid eutectic mixture with the incorporation of silica nanoparticles at 0.5% wt., 1% wt., and 1.5% wt. The authors characterized the thermal stability of the enhanced material through differential scanning calorimetry and thermogravimetric analysis. It was evaluated for its long-term thermal performance after 2000 cycles by thermal stability tests. The authors concluded that the nano-enhanced phase change material was thermally stable within their working temperature range and will ensure a long-term performance within the application temperature ranges of the residential buildings. The homogeneity and long-term stability of the nano-enhanced phase change materials with base molten salts without agglomeration and sedimentation of the nanoparticles are also vital issues to be addressed. The high surface area, surface charge, and van der Waals forces of the nanoparticles lead to their sedimentation and non-homogeneous dispersion, and this could negatively affect the thermophysical properties of the inorganic molten salts. The stability of the nano-enhanced phase change materials is usually evaluated by visual inspection for prolonged periods. Also, many routes can be followed to change the surface of the nanoparticles to prevent agglomeration and improve their stability in molten salts such as surface modification, addition of surfactants, sonication, and pH adjustment. A surfactant can be used during the synthesis of the nano-enhanced phase change materials to improve the stability over time of the nanoparticles in the salt, particularly the hydrophobic ones, by reversing their behavior while preparing the enhanced materials by the wet method. The surfactants adhere to nanoparticles and change their wettability and the surfactant content should be carefully controlled, given that an insufficient or excessive amount of surfactant would affect the forces between the nanoparticles—zeta potential or van der Waals—and impact the dispersion uniformity of the nanoparticles. The surfactants used with molten salts should also withstand temperature values greater than 450 °C, and examples of employed surfactants are sodium dodecylbenzene sulfonate, sodium dodecyl sulfate, and gum arabic. Additionally, the ultrasonication method is used to deagglomerate the clusters of the nanoparticles without altering their surface characteristics. The researchers Chen et al. [94] found a specific heat decrease of 8.5% after 200 h at a high temperature for nano-enhanced phase change materials prepared by the wet method and a 5% reduction after 2000 h for nano-enhanced phase change materials prepared by high-temperature melting. The authors noted that the materials produced using ultrasonication could not withstand high temperatures for long periods. Nonetheless, the ultrasonication methodology is still considered to be the best way to deagglomerate the nanoparticles and result in homogeneous dispersions in molten salts. Moreover, developing a homogeneous molten salt nano-enhanced phase change material, while maintaining the stability of nanoparticles after several cycles of high-temperature heating and cooling, is a challenge that should be attained. In the surface modification approach, surface functionalization is utilized to change the surface of the nanoparticles, aiming to reduce their surface energy by covalent coupling, physical adsorption, and electrostatic binding. This method improves the stability, without compromising the thermophysical characteristics of the molten salt base fluid, which may be collateral damage imposed by the previously described techniques. The stability of the nano-enhanced phase change materials decreases when the pH is close to the isoelectric point. The tendency of the nanoparticles to agglomerate increases as the repulsive force between them, which is directly affected by the pH, decreases [95]. Also, the researchers Mondragon et al. [96] measured the pH of lithium, sodium, and potassium nitrates with the addition of silica and alumina nanoparticles. The authors found that the pH of the nano-enhanced phase change materials was less than that of the isoelectric point, providing enhanced dispersions and extended stability of the nanoparticles. Nonetheless, there is no published work on the stability of nano-enhanced phase change materials after being heated to high temperatures, given that the bond between the added surfactant and the nanoparticles could be affected at temperatures greater than 60 °C [97]. It should be highlighted that more research studies are needed to better understand the impact of the pH adjustment on the thermophysical properties of the molten salt nano-enhanced phase change materials.

## 4. Phase Change Influencing Factors

The phase change of the nano-enhanced phase change materials depends primarily on the phase change temperature, pressure, and period in which the transition of phase occurred, and other factors like the elastic modulus. If the phase change materials are emulsions, the fundamental factors that affect their phase change are the water content, temperature, salinity degree, pH, shear rate, and time, among others. Also, the phase change hysteresis phenomenon of the phase change energy storage materials should be considered, as it has a significant impact on the charging and discharging performance of phase change materials. The hysteresis characteristics of phase change energy storage materials are based on the fact that the temperature range of phase change of the energy storage materials is different in the process of heat storage and heat release, and there is a difference between melting temperature and crystallization temperature. The phase change hysteresis is a common phenomenon in the phase change process, which has a great influence on phase change materials. The phase change materials can be divided into liquid–gas phase, solid–gas phase, solid–solid phase, and solid–liquid phase according to their phase change mechanism, but a major part of them have the solid-to-liquid melting phase and the liquid-to-solid solidification phase. The phase transition hysteresis can be defined by the difference between the actual melting point and freezing point of the phase change materials. The thermal hysteresis of many materials arises from the subcooling or superheating effects, which should be carefully considered in the phase change materials for renewable energy systems where the temperature differences are usually very small. Moreover, some published works have demonstrated that in the exothermic process of phase change materials, the phase change temperature in the solidification stage was influenced by the final formulation of the included nanomaterials, nucleating agents, and additives, latent heat, and thermal diffusivity, which could be the fundamental factors causing undercooling and phase change hysteresis [98]. Additionally, the researchers Sandy et al. [99] investigated the influence of thermal hysteresis on the design factors of thermal energy storage systems. The accuracy of the results was investigated and the impact of alterations in the design factors and capsule material with thermal hysteresis in spherical phase change material capsules was addressed. The authors stated that the phase change material having high thermal hysteresis made the accuracy of the melting time and operating temperature low. Also, the researchers Moreles et al. [100] conducted a numerical study of the combined effects of phase change hysteresis in residential buildings’ phase change material envelopes. The authors developed a code for simulating hysteresis that employed a specific heat method to simulate the heat transfer process through the phase change material. The authors argued that the thermal hysteresis improved the thermal performance of the phase change material envelopes with a greater hysteresis temperature difference. Furthermore, the authors Delcroix et al. [101] obtained the enthalpy vs. temperature and specific heat vs. temperature curves of phase change materials under different methods and heat transfer rates. The authors found that the phase transition hysteresis was closely linked to the heat transfer rate, and the higher the heat transfer rate, the greater the temperature difference of the phase transition hysteresis. The researchers Hsu et al. [102] studied three different metal microcapsules phase change materials composed of zinc–titanium oxide, zinc–alumina, and zinc–silica. The obtained results showed that the phase change microcapsule materials had a large phase change lag, and the phase change lag increased with increasing thickness of the shell and temperature difference. It should be concluded that the melting and solidification points, phase change latent heat, material density, and thickness impact the phase change hysteresis. Also, the encapsulation method can alter the thermal performance of the phase change materials concerning the phase transition temperature and phase transition hysteresis. 

## 5. Characterization of the Nano-Enhanced Phase Change Materials

Nano-enhanced phase change materials are characterized by a set of suitable techniques to study their nanostructures, compositions, thermal characteristics, and phase change parameters. One such technique is scanning electron microscopy, which enables the study of the nanostructures formed in the adjacent areas of the incorporated nanoparticles and the base phase change material (e.g., molten salts) to better comprehend the underlying mechanisms of the improvement/deterioration of the thermophysical properties of the enhanced materials. Also, scanning electron microscopy can closely evaluate the degree of homogeneous dispersity of the added nanoparticles and the existence of agglomerates. Finally, this technique enables researchers to determine the size of the nanoparticles added to the nano-enhanced phase change materials. Additionally, the energy-dispersive X-ray spectrometry technique is usually applied to study the elements and respective concentrations by bombarding the nano-enhanced phase change material with an electron beam and analyzing the emitted X-ray spectrum to identify the existing elements. X-ray diffraction techniques are employed to verify whether a chemical reaction occurred between the base phase change material and the nanoparticles by the detection of new material formation. Additionally, X-ray diffraction is also used to examine the corrosion products of the nano-enhanced phase change materials after corrosion tests. The Fourier transform–infrared spectroscopy technique is employed to obtain the infrared spectrum of absorption or emission of the nano-enhanced phase change materials. The infrared absorption spectroscopy region is between 4000 cm^−1^ and 400 cm^−1^, which is the absorption radiation of most organic and inorganic ions. Infrared absorption spectroscopy is applied to analyze the structures of the existing molecules to identify the components in the enhanced material and the amount of each component in a mixture based on the absorption of specific wavelengths of infrared radiation by the molecules. For example, the researchers Mondragon et al. [96] used Fourier transform–infrared spectroscopy to relate the ionic exchange capacity of the nanoparticles to the improvement in the thermophysical properties. Some researchers used this technique to investigate the prospect of the degradation of the nano-enhanced phase change materials after a corrosion test [103]. The nano-enhanced phase change materials are usually analyzed by differential scanning calorimetry to determine their specific heat, melting point, and enthalpy. This technique detects the temperature gradients between the material and reference by heating them at the same heating rate until the tested material undergoes a phase transition and absorbs or releases heat. More heat will flow into the sample to maintain a temperature similar to that of the reference. The phase change temperature and heat capacity are determined by measuring how much more or less heat the material needs to maintain its temperature at that of the reference. The thermogravimetric analysis is a technique that can be explored to determine the thermal stability of a material by measuring the alterations in the weight of the material as a function of temperature or time. If the material is thermally stable, there will be no modification in its mass. As the material is heated, it starts to lose weight. The weight decrease is plotted as a function of the temperature, and the onset of the plot is used to determine the decomposition temperature. Moreover, the thermogravimetric analysis can be used to measure the maximum operating temperature of the nano-enhanced phase change materials. The dynamic viscosity of the nano-enhanced phase change materials in the molten state can be evaluated by using a rheometer controlling the shear rate, or alternatively, the shear stress. Finally, the corrosivity of the nano-enhanced phase change materials can be measured by dynamic or isothermal immersion tests. Usually, a representative sample of the concentrated solar power piping or tank materials is weighed and immersed in nano-enhanced phase change material for a well-defined period. After that, the sample is again weighed to determine the weight reduction and infer the corrosion rate. For more examples of research studies using the mentioned characterization techniques, one may refer to the preparation of myristic acid–palmitic acid–stearic acid with the addition of expanded graphite nano-enhanced phase change material by the authors Yang et al. [104]. The mass fraction of the fatty acid mixture used was around 93% wt. The properties and the microstructure of the nano-enhanced phase change material were obtained by differential scanning calorimetry, Fourier transformation infrared spectroscopy, thermogravimetric analysis, and scanning electron microscopy. The authors reported that the melting temperature and latent heat were around 42 °C and 153.5 kJ kg^−1^, respectively. Also, the authors Pielichowska et al. [105] studied the synthesis of the polyurethane-based phase change material containing poly(ethylene glycol), 1,4-butanediol, and 4,4′-diphenylmethanediisocyanate with the addition of different concentrations of graphite nanoplatelets. The authors prepared the composite phase change materials with and without the addition of 1,4-butanediol, which was employed to extend the chains. The research team found that the latent heat of fusion and the melting temperature of the composite phase change materials were in the range of 118–164.5 kJ kg^−1^ and 53.1–66.6 °C without butanediol and 128–148.5 kJ kg^−1^ and 50.2–63.8 °C with butanediol. They applied Fourier transform–infrared spectroscopy to obtain representative spectra of the nano-enhanced phase change materials. These spectra presented the characteristic absorption bands for the polyurethane–polyethylene glycol and polyurethane–polyethylene glycol with 1,4-butanediol systems having different concentrations of graphite. Moreover, the authors Karaipekli and Sari [106] used the capric–lauric, capric–palmitic, and capric–stearic acid eutectic mixtures to prepare nano-enhanced phase change materials with the addition of expanded vermiculite. Vermiculite, a clay mineral also known as hydrated laminar magnesium–aluminum–iron silicate, was employed as an additive to obtain a more stable form of the developed materials. The latent heat and melting temperatures of the nano-enhanced phase change materials were between 61 kJ kg^−1^ and 72 kJ kg^−1^ and between 19 °C and 25.6 °C, respectively, and their determination was based on differential scanning calorimetry analyses. Finally, more nano-enhanced phase change materials were characterized by the authors Mullangi et al. [107,108], and Evans et al. [109] used many techniques to characterize their nano-enhanced phase change materials. 

## 6. Thermophysical Properties Modeling

**Figure 9:**
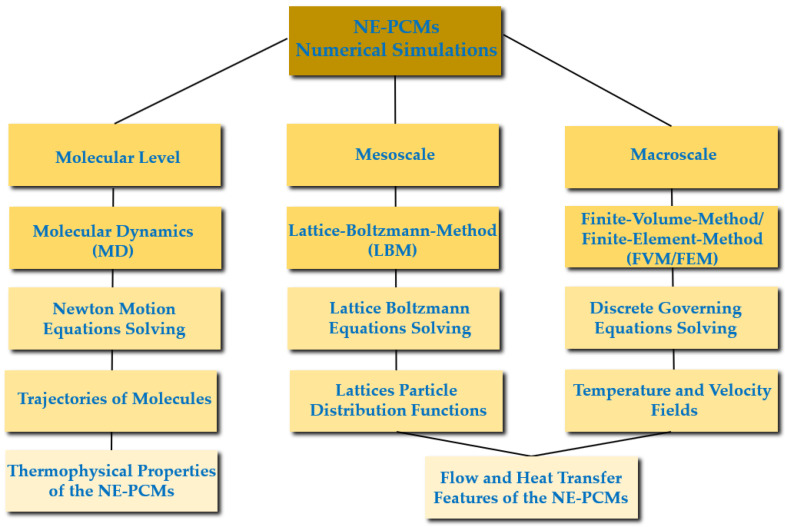
Main numerical simulations routes of the nano-enhanced phase change materials.

The application of nanoparticles as a phase change material enhancer was first numerically studied by the authors Khodadadi and Hosseinizadeh [110]. The authors numerically investigated the general impact of adding copper nanoparticles into water during the freezing process. The researchers considered the single-phase model assuming a homogeneous distribution of the incorporated nanoparticles in the base fluid. The obtained predictions showed that a higher heat release rate and thermal conductivity can be attained compared to those achieved with the water itself. In previous research works, the considered mixture theoretical model was the single-phase one with governing equations for the specific heat, latent heat, thermal expansion coefficient, and density. The authors Vajjha and Das [111] proposed empirical correlations for the thermal conductivity and viscosity of the nano-enhanced phase change materials. The thermal conductivity correlation included the contribution of the morphology, concentration, and Brownian motion of the nanoparticles. Furthermore, the researchers Gunjo et al. [112] numerically investigated the incorporation of 5% wt. of copper, copper oxide, and alumina nanoparticles in paraffin. The authors employed the single-phase model not considering the agglomeration and sedimentation processes and the thermophysical properties were assumed to be homogeneous and isotropic. The investigation team found that the melting time of the paraffin was 10 times, nearly 4 times, and around 2 times faster with the addition of copper, copper oxide, and alumina nanoparticles, respectively. At the same time, it was found that the solidification time exhibited an 8-fold, 3-fold, and nearly 2-fold increase in the latent heat storage with the inclusion of copper, copper oxide, and alumina nanoparticles, respectively, in comparison to the latent heat storage obtained with only the paraffin. Also, the authors Iachachene et al. [113] examined the nano-enhanced phase change material composed of paraffin wax and graphene nanoparticles. The research team observed that the heat transfer behavior was substantially ameliorated, and the thermal conductivity was increased by up to 80% compared to that attained with the paraffin wax itself. Furthermore, the authors Gorzin et al. [114] evaluated the solidification process of a phase change material with the addition of copper nanoparticles operating in a heat exchanger. The authors applied the single-phase change model and estimated the effective thermal conductivity and viscosity through empirical correlations. The obtained results revealed a solidification duration reduction of 8% at a concentration of 2% wt. and of 15% at 4% wt. The results also indicated that the thermal conductivity of the synthesized enhanced material was increased, and its latent heat capacity was decreased. Additionally, the researchers Kheshteli and Sheikholeslami [115] examined the solidification rate using nanoparticles and wavy triplex tubes. The authors used numerical predictions that proved that the geometric modifications of tubes having wavy shapes may result in a shorter solidification process time. Also, it was confirmed that the combined use of the wavy-shaped tubes and nanoparticles led to an improved heat transfer performance. Consequently, increasing the concentration of nanoparticles up to 5% vol. decreased the solidification duration by 20%. Once more, the authors employed the single-phase model with mixture relations for the specific heat, latent heat, and density and used the same empirical correlations for the thermal conductivity and viscosity as those adopted by the researchers Khodadadi and Hosseinizadeh [110] and Harikrishnan et al. [47]. These authors evaluated the impact of multicycle processes on the thermal energy storage capability of stearic acid with the addition of titanium oxide nanoparticles. The researchers observed that, over 5000 cycles, the maximum alterations of the melting and solidification points were −0.35 and −0.6%, respectively. Also, over the same number of cycles, the maximum alteration of the latent heat capacity was nearly 1.3% and 1.5% for the melting and solidification processes, respectively. In addition, the authors confirmed that the melting time was reduced by nearly 43.7% and the solidification time was reduced by nearly 41.4% at 0.3% wt. of nanoparticles. Accordingly, the authors Harikrishnan et al. [47] found similar results for the case of myristic acid with the incorporation of silica nanoparticles. Consequently, considering the previous studies, it can be concluded that nano-enhanced phase change materials are an attractive choice to overcome the slow response of thermal energy storage systems during their charging and discharging stages. In addition, if optimized designed containers are filled with nano-enhanced phase change materials, the improvement would be even greater. Nonetheless, all the numerical studies carried out to date adopted the single-phase model in which the Brownian diffusion, thermophoresis diffusion, and sedimentation of the nanoparticles are not considered. Indeed, the single-phase model has been confirmed to not possess an accurate estimation of the heat transfer enhancement magnitude, specifically at nanoparticle concentrations greater than 1% wt. [116]. Also, the single-phase model is not capable of predicting the nano-enhanced phase change materials’ behavior during the charging and discharging cycles. Hence, the single-phase model hardly provides exact outcomes from the phenomena associated with the thermal energy storage systems operating with these materials for extended periods. Such facts derive from the assumption of a homogeneous distribution of nanoparticles within the base fluid and, consequently, the results of a multi-cycle numerical simulation would be meaningless, given that the predictions of the consecutive cycles will be equal. A two-phase model would be more suitable for the multi-cycle simulations considering the Brownian diffusion and thermophoresis diffusion slip mechanisms, as proposed by the author Buongiorno [117], then further numerically ameliorated by the authors Riahi et al. [118] and finally upgraded by Amidu et al. [119] to also consider the sedimentation of the nanoparticles. Therefore, it is suggested to follow the advanced two-phase model of Amidu et al. [119], considering the three slip mechanisms of the nanoparticles, combined with an enthalpy–porosity method to achieve an improved understanding of the different mechanisms involved in the enhanced materials’ thermal behavior. It should be stated that the two-phase model includes the slip motions of the nanoparticles in the phase change materials caused by sedimentation, thermophoresis, and Brownian diffusion. Previous investigations of nano-enhanced phase change materials often adopted the single-phase model, which becomes inadequate when such materials operate over many charging/discharging cycles. When these materials operate for a single charging/discharging cycle with a homogeneous distribution of nanoparticles, a considerable improvement in the heat transfer performance was observed relative to that of the base phase change material itself. Such evidence is consistent with the findings exposing distinct levels of enhancement. Nonetheless, the continuous operation of the enhanced materials over more than one charging/discharging process has shown a significant deterioration of the heat transfer behavior provoked by the non-homogeneity of the nanoparticles concentrations due to their migration motion caused by their sedimentation, Brownian diffusion, and thermophoresis, resulting in the deposition over time of the nanoparticles. This deterioration of the heat transfer behavior at concentrations greater than 1% wt. is exploited by the two-phase model in which the nanoparticle migration mechanisms of importance are considered. A comparison of the first and second charging involving the nano-enhanced phase change materials with solely the first charging of the pure phase change material showed that the performance of the enhanced materials at the second charging was appreciably reduced by nearly 50%, better than the performance of the pure phase change material, which was reduced by around 20%. Therefore, it can be concluded that the investigation of the nano-enhanced phase change materials following a single-phase model over an individual cycle of charging/discharging does not provide a complete view of the heat transfer behavior of such materials. Rather, a two-phase model that accounts for the nanoparticle slip underlying mechanisms is proposed for a more complete investigation of the thermal performance of the nano-enhanced phase change material operated over several charging/discharging cycles. Figure 9 shows the fundamental scales, methods, and outcomes of the numerical simulations of the nano-enhanced phase change materials.

## 7. Metal–Organic Frameworks and Covalent Organic Frameworks

Metal–organic frameworks (MOFs) are crystalline materials composed of metal ions or metal clusters and organic linkers [120]. As an innovative type of porous material, MOFs have attracted great attention in recent years owing to their high surface area and permanent porosity. MOFs are made by linking inorganic and organic units via strong chemical bonds. The organic units are divalent or polyvalent organic carboxylates, which, when linking to metal-containing units (e.g., Zn2þ, Cu2þ, Mg2þ, Al3þ), can yield three-dimensional structures having well-defined pore size distributions. The surface area of metal–organic frameworks ranges from 1000 to 10,000 m^2^/g, and the pore size can be tuned by altering the organic and metal-containing units [121]. Transition metals including zinc, copper, and iron, alkaline earth elements, p-block elements like indium and gallium, actinides, and mixed metals have been used to produce MOFs [122]. The most prevailing synthesis methods for MOFs are hydrothermal and solvothermal approaches [123]. In a typical solution-based MOF process, a nanoporous material can be formed by nucleation and spreading, and then multiple nucleations aggregate with surface-adsorbed organic molecules into a mixed inorganic–organic crystal. To produce controllable nanoscale MOFs crystals and shorten the synthesis time, some alternative fabrication methodologies have been followed, including sonochemical [124] and microwave-assisted synthesis [125]. By observing MOF surface characteristics in the nucleation and spreading processes, the authors Moh et al. [126] developed the preparation method of Zeolitic Imidazolate Framework-8 (ZIF-8). The porous ZIF-8 was obtained by the nucleation and spreading of successive surface steps of the enclosed framework structure. It should be highlighted that because of the adjustable porous nanostructures, MOFs are now considered one of the most promising alternatives to be applied in energy storage and conversion processes. Besides MOFs, there are MOF-derived formulations with adjustable nanostructures, which have received great research interest for electrochemical applications. Also, since MOFs can be converted by one-step calcination, they have been applied to synthesize nanostructured carbon, metal oxides, and metal oxide–carbon nanocomposites. The large amount of carbon-based organic linkers in MOFs aids in the development of nanostructured carbon with MOFs as sacrificial materials. Because MOFs are composed of metallic species, it is vital to remove such species to obtain high-surface-area carbon. Another way to synthesize high-porosity nanostructured carbon and obtain high-surface-area carbon is to perform the infiltration of a secondary carbon source into the MOFs [127]. Accordingly, the authors Xu et al. [128] obtained high-surface-area carbon by introducing furfuryl alcohol into pristine ZIF-8 as a secondary carbon source, attaining a considerably high surface area of 3405 m^2^g^−1^ and a total pore volume of around 2.6 cm^3^g^−1^. Also, other carbon-based organics such as glycerol, carbon tetrachloride, and ethylenediamine have been explored as secondary carbon sources introduced in the cavities of the MOFs for synthesizing nanostructured carbon. Because of the structures of the MOFs that are made of metal centers coordinated with organic complexes, the controlled heating in different environments may synthesize MOF-derived metal oxide structures. Moreover, the researchers Ma et al. [129] utilized cobalt MOFs to produce Co–Nx–Carbon nanoparticles. During the thermal carbonization, the organic linkers were converted to carbon, whilst keeping a porous framework, with cobalt–Nx units uniformly dispersed in the carbon framework. The MOFs can act as electrode catalysts or catalyst precursors by adjusting their structures to obtain the optimized materials for fuel cells. As fuel cell catalysts, copper MOFs, iron MOFs, and MOF–graphene composites possess enhanced catalytic activity and stability. By selecting metal clusters and multi-functional linkers, the MOFs can be developed with rich transition metal–nitrogen–carbon sites, which would be advantageous for producing improved electrochemical catalysts. Nonetheless, the synthesis of highly electrically conductive MOFs is still a considerable challenge when applied to MOFs for energy storage and conversion purposes. Also, the achievement of controllable structure and morphology of MOF-derived porous carbon or metal oxide composites still requires further research, as the uniform distribution of metal particles in a carbon matrix is still not an easy task to accomplish. The authors Maity et al. [130] published meritorious findings about MOF development. Covalent organic frameworks (COFs) are a class of polymers that enable the predesign of both primary- and high-order structures and the synthesis of long-range structures through one-pot polymerization. Progress over the past decades in chemistry has dramatically enhanced our capability of designing and synthesizing COFs and deepening the understanding of exploring energy-converting functions originating from their ordered skeletons and channels. COFs are formed via the polymerization of monomers in which the chain propagation is controlled by a topology diagram so that each bond connection is spatially confined in either a two- or three-dimensional way or geometrically guided to produce extended and ordered two-dimensional or three-dimensional polymer architecture. The geometry and backbone of the organic units predetermine the dimensionality, along with the shape and size of the polygons in COFs. During polymerization, the two-dimensional network crystallizes to form two-dimensional layer frameworks induced by interlayer π–π stacking interactions. Two-dimensional COFs offer ordered columnar π-arrays, as well as aligned one-dimensional nanochannels. The ordering structure of the COFs involves the periodically arranged skeleton and the aligned channels. Owing to these features, COFs offer an irreplaceable platform for developing polymers to achieve the desired skeleton and pore structure. The above-mentioned characteristics of COFs are inaccessible with traditional polymers and porous materials, whereas the previous definition of the skeleton and channel enables the designing of structures and functionalities for energy conversion systems. Also, COFs clearly offer the opportunity for engineering organic materials to confront environmental and energy concerns. Nonetheless, COFs’ development is in the embryonic stage, as there are still many issues to better understand. The formation of defects and their mechanisms are still not well understood. Also, less is understood regarding the defects and their three-dimensional distributions in the framework. This is a subject that connects with the preparation of high-quality crystallites of COFs. A diversity of reactions has been developed for the synthesis of COFs. However, several of them are very limited in terms of crystallinity, monomer scope, and reaction conditions. Exploring the reactions to form stable crystalline porous frameworks is still an appreciable challenge. In this case, the crystallinity and porosity need to be considered, as materials of low crystallinity and porosity hardly present the inherent properties of a COF with the desired structural integrity and functionality. Studies on these basic issues can disclose the nature of COFs both in structure and property. There is an increasing trend for laboratory work on the properties and functions of COFs when designed for different purposes. Nonetheless, the published works have not yet identified the COFs’ features owing to their low crystallinity and porosity. In relation to energy conversion, COFs have shown considerable advantages in designing π-architectures via the merging of π-arrays with catalytic sites and built-in channels. Specifically, COFs’ polymerization under topology guidance enables the integration of various organic blocks into well-defined polymer backbones to achieve long-range-ordered structures, which opens an avenue to design catalysts for energy conversion. The alignment of the components and linkages defines the possibility of obtaining HOMO and LUMO levels, band gaps, catalytic sites, redox potentials, and diverse interfaces using one framework. Such features make COFs very suitable for exploring catalysts to increase energy conversion capability. Also, in terms of photocatalytic conversion, the use of catalytic sites for the reduction and oxidation of water into hydrogen and oxygen is a crucial issue. Although it was already demonstrated the potential of COFs to be used in photocatalysis other than common platinum nanoparticles and iridium complex, most cases focus on the performance evaluation without the desirable identification of active sites, mechanisms, and catalytic cycles. For instance, in the photocatalytic reduction of carbon dioxide, the noble metal-free and sacrificial electron donor-free arrangements are of critical importance. Concerning the electrocatalytic reduction of carbon dioxide, COFs demonstrated great potential for combining conductivity with catalytic activity. Building blocks with diverse reactive sites were connected via covalent bonds to construct COFs with various pore structures based on topology diagrams. The combination of building blocks with specific geometries generated COF backbones with specific polygonal structures that grew repeatedly to form COFs with periodic structures. The COFs are designed by combining one knot and one linker, in which the knots represent branched building blocks to extend the framework, while the linkers are employed to connect the knots. The multi-component strategy with knots and linkers can prepare COFs with complicated backbones and irregular pore structures, which significantly expanded the complexity and diversity of COF structures [131]. The three-dimensional COFs were constructed by introducing TD (*endo*-tricycle [4.2.2.0] deca-3,9-diene) or orthogonally symmetric blocks into three-dimensional frameworks. The combination of the TD monomers with C2, C3, and C4-symmetric monomers, or TD monomers enables the design of three-dimensional COFs [132]. Significant efforts focusing on the diversity of COFs, including building blocks, linkages, reactions, and functionalities toward photocatalysis, have already been made. These achievements have made COFs a promising platform for photocatalysis processes. Nevertheless, many fundamental issues and challenges still need to be addressed and resolved for the further development of COFs, from laboratory research to commercial and industrial applications. The photocatalytic activities of COFs and their composites are highly dependent on the chemical structures of the monomers and linkages, which can precisely modulate the photophysical and photochemical properties of the materials. The basic principles for the exploration of novel building blocks and linkages toward COF photocatalysts include improving the visible light absorption ability, enhancing photogenerated charge carrier transportation and separation efficiency, and inhibiting the recombination of holes and electrons. For the design of building blocks, the development of photoactive monomers and the formation of donor–acceptor structures will enable the control of the optical band structures of photocatalysts and facilitate electron–hole pair transfer and separation. For the design of linkages, the fully conjugated linkages such as carbon–carbon double bonds and phenazine have superior photonic and electronic conductivity as compared to the boronate ester and imine linkages. The exploration of COF photocatalysts having conjugated linkages has great potential for boosting photocatalytic activities. The crystallinity of COFs is a crucial factor impacting the mobility and lifespan of the photo-induced charge carriers that can be modulated by pre-designing suitable monomers and linkages. A deep investigation of the mechanism and nature of the fundamental steps involving photocatalysis at the molecular level remains elusive because of the defects and disorders in COF polycrystalline powders. The authors Mullangi et al. [107,133] and Chakraborty et al. [134] published meritorious works on the development of COFs. 

## 8. Main Applications of Nano-Enhanced Phase Change Materials

### 8.1. Historical Background of Nano-Enhanced Phase Change Materials in Energy Applications

Phase change materials are very suitable for storing energy in the form of heat, whenever solar energy is available. Nevertheless, these materials exhibit properties such as fast charging and releasing and demonstrate high thermal performance. However, such materials should be used in photovoltaic/thermal systems with higher temperatures during sunshine and lower warmth at nighttime to attain heat removal and higher solidification for regeneration. Organic and inorganic phase change materials are commonly used for storing heat energy. Many authors have proposed dispersing nanoparticles into the base phase change materials, the encapsulation of phase change materials with nanoparticles, and the use of nano-enhanced phase change materials, since these methodologies will improve the thermal performance of the phase change materials in energy harvesting and conversion processes. The nano-enhanced phase change materials not only increase the thermal conductivity but also modify properties like latent heat, density, specific heat, and thermal reliability. The authors Li et al. [135] argued that the addition of spongy graphene in docosane phase change material improved its thermal conductivity by more than twofold and its latent heat by 62%. Moreover, the researchers Al-Jethelah et al. [136] reported that using metallic foam and copper oxide nanoparticles in coconut phase change materials provided an ameliorated energy storage capability. The authors stated that the energy storage capability was increased by approximately 29%. Additionally, the researchers Babapoor et al. [85] performed experimental work on the addition of nanoparticles in a base phase change material and reported modifications in their properties. The research team also noticed that the addition of nanoparticles into the phase change material altered the thermal conductivity, latent heat, dynamic viscosity, phase transition temperature, and time. Also, the authors Masoumi et al. [137] investigated the use of carboxylic acid and titanium oxide, and a nano-enhanced phase change material was produced by spreading titanium oxide nanoparticles in stearic acid at mass fractions varying from 0.09% wt. to around 0.4% wt. The prepared nano-enhanced phase change material had a thermal conductivity increase of 27% at 0.36% wt. The developed enhanced material was thermally stable after 250 thermal cycles and, consequently, could be suggested for solar thermal energy storage applications. Also, it was found that titanium dioxide helped in maintaining the latent heat due to its porosity. Furthermore, the authors Sivasamy et al. [138] evaluated the thermal conductivity of silver nanoparticles dispersed in myristic acid. The thermal conductivity increases at 0.1% wt., 0.2% wt., and 0.3% wt. were near 42%, 72%, and 109%, respectively, compared to the pure myristic acid. The researchers argued that the high surface area enabled a faster phonon movement when the silver nanoparticles were added to the myristic acid. The melting point at 0.3% wt. was around 56 °C, which was slightly higher than that of the myristic acid, and it was due to the bonding of the acid molecules with the silver nanoparticles and the retention of acid in the porous nanostructure of the silver particles. Also, the latent heat for the best nano-enhanced phase change material was around 190 J/g, which was slightly lower than the latent heat of the myristic acid. The authors explained this by the addition of the nanoparticles and the high melting temperature. The enhanced composite having different proportions of silver nanoparticles was suitable for low-temperature solar power thermal energy applications. In addition, the researchers Song et al. [139] prepared a nano-enhanced phase change material composed of the base polyethylene glycol phase change material and 1D halloysite nanotubes assembled with silver. The nanostructure assembled with silver nanoparticles was produced by chemical reduction and self-assembly procedures. The thermal conductivity of the prepared material was 0.9 W/m.K, which was around 2 times greater than that of the polyethylene glycol. The prepared material melted at around 34 °C and solidified near 26 °C with a phase change heat of 71 J/g during the melting process and 68 J/g during the solidification. The nano-enhanced phase change material retained its thermal stability after 2000 thermal cycles. The porosity of silver nanoparticles helped prevent the leakage of the phase change material, thereby maintaining the latent heat for thermal energy storage. Also, the researchers Zhang et al. [13] developed a sunlight-driven nano-enhanced phase change material made of polyethylene glycol, silver, and graphene nanosheets. The material showed thermal conductivity increases between around 50% and 95%. The thermal conductivity of the material was increased to approximately 0.4 W/m.K due to the inclusion of graphene nanosheets and silver nanoparticles. The increased surface area of the graphene nanosheets resulted in more thermal conductivity enhancement in the phase change material. The ideal melting temperature of the material was approximately 60 °C, which was slightly lower than that of the pure phase change material. The phase change material shape stability was explained by the authors as being the result of the abundant wrinkles on the surface of the silver–graphene nanoplatelets. The researchers Li et al. [140] prepared sodium acetate trihydrate salt and porous copper foam to mitigate the phase separation and supercooling limitations. The thermal conductivity of copper foam–sodium acetate trihydrate-enhanced phase change material was 11 times higher than that of sodium acetate trihydrate. The thermal conductivity increase was explained by the researchers as being based on the decrease in the porosity of the copper foam that, in turn, increased the phonon motion. The prepared enhanced material showed good phase separation at 0.5% wt. of carboxyl methylcellulose and 2% wt. of disodium hydrogen phosphate dodecahydrate. The melting onset temperature was 58 °C, which was closer to the melting point of the sodium acetate trihydrate. The nano-enhanced phase change material exhibited improved thermal stability and supercooling of less than 3 °C. The copper foam helped maintain the required latent heat for solar thermal storage. The thermal conductivity increase affects the thermal energy storage capability of the nano-enhanced phase change material since a greater number of nanoparticles will decrease the phase change material amount. The nano-enhanced phase change material had good thermal conductivity and could be employed in faster heat storage/release places. Furthermore, the researchers Xiao et al. [141] produced a nano-enhanced phase change material by mixing sodium acetate trihydrate with xanthan gum and copper foam through vacuum impregnation. The thermal conductivity of the produced material was 2.1 W/m.K, which was almost two times greater than that of sodium acetate trihydrate with xanthan gum, indicating that the increased thermal conductivity was due to the presence of the copper foam. The sodium acetate trihydrate with xanthan gum melted at around 59 °C, which was a value higher than the sodium acetate trihydrate temperature of melting. This was maybe due to the bonding between the phase change materials and additives. The material was found to be thermally stable after 200 thermal cycles. Copper is an excellent heat conductor and when coupled with other materials, it increases their thermal conductivity. The high thermal conductivity and latent heat make it suitable in places where the thermal energy storage processes occur at a faster phase. The researchers Sheng et al. [142] prepared a nano-enhanced phase change material composed of erythritol and aluminum. The material had a thermal conductivity of 30 W/m.K at around 42% vol. of aluminum. The authors argued that the agglomeration of the aluminum nanostructures was one of the main factors impacting the thermal conductivity increase. The addition of nanoparticles could ensure that the latent heat did not deteriorate. The high thermal conductivity along with the good latent heat storage make the prepared material a good candidate for the rapid heat storage and release required in solar thermal applications. Additionally, the researchers Wei et al. [143] synthesized a nano-enhanced phase change material having alumina with a supporting matrix and a eutectic mixture of lauric acid–stearic acid–myristic acid as a base phase change material. The thermal conductivity of the developed material was higher than that of the base phase change material. The porous structure of the alumina facilitated the phase change material’s confinement, leading to increased heat transfer and effective melting. The latent heat of the nano-enhanced phase change material was 113.7 J/g during the melting process and 108.5 J/g during the solidification process. The melting and solidification temperatures were 28.6 °C and around 27 °C, respectively. As the incorporated alumina was porous, it kept the latent heat of the phase change material. The developed material was thermally stable and chemically inert, and had good thermal conductivity, adequate to be used in solar thermal applications. The porosity helped the retention of the phase change material and maintained the latent heat while charging/discharging. Furthermore, the researchers Singh et al. [144] studied the thermophysical characteristics of myo-inositol with the addition of alumina and copper oxide nanoparticles for developing a more efficient solar thermal energy storage system. The results showed that the myo-inositol-based nano-enhanced phase change material was very suitable to be applied in solar thermal energy storage processes at temperatures from 160 °C to 260 °C. Also, the copper oxide nanoparticles were reported to be more suitable for this purpose as compared to the alumina ones due to their increased melting point. Moreover, the authors Sharma et al. [43] proposed a passive cooling solution for residential buildings’ integrated concentrated photovoltaics by incorporating micro fins, phase change materials, and nano-enhanced phase change materials. The results showed that the temperature at the center of the system was reduced by around 11 °C when using micro fins and phase change materials and by 12.5 °C when using micro fins and nano-enhanced phase change materials. Temperature reductions of 9.6 °C and 11.2 °C were verified in the cases where the phase change materials and nano-enhanced phase change materials were employed with un-finned surfaces as compared to natural convection. The results showed that the combined usage of passive technologies could be a reasonable solution for the thermal performance enhancement of the concentrated photovoltaics of intelligent buildings. The researchers Abdelrahman et al. [145] experimentally studied the thermal performance of photovoltaic cells employing alumina nanoparticles dispersed in the RT35HC thermal fluid. Also, the alumina nanoparticles at concentrations varying from 0.11% vol. to 0.77% vol. were mixed with a phase change material, and cylindrical fins were utilized as heat sinks. The results showed a front surface temperature reduction of between 20% and around 46% in the case of using the phase change material and cylindrical fins. The temperature reduction was enhanced to around 52% with the addition of the nanoparticles. Also, the authors Zarma et al. [146] explored the performance enhancement of a concentrator photovoltaic nano-enhanced phase change material hybrid system with the addition of alumina, copper oxide, and silica nanoparticles at concentrations from 1% wt. to 5% wt. into CaCl_2_·6H_2_O. The results indicated considerable thermal conductivity enhancements when using alumina nanoparticles as compared to copper oxide and silica ones. The alumina enhanced the electrical efficiency of the system by 8%, which was greater than that attained with the pure phase change material (6.3%). Zeng et al. [147] dispersed silver nanoparticles in 1-tetradecanol phase change material for thermal conductivity enhancement purposes. The obtained results showed that the thermal conductivity of the base phase change material increased with increasing concentrations of silver nanoparticles. 

### 8.2. Solar Thermal Energy Storage Systems

**Figure 10:**
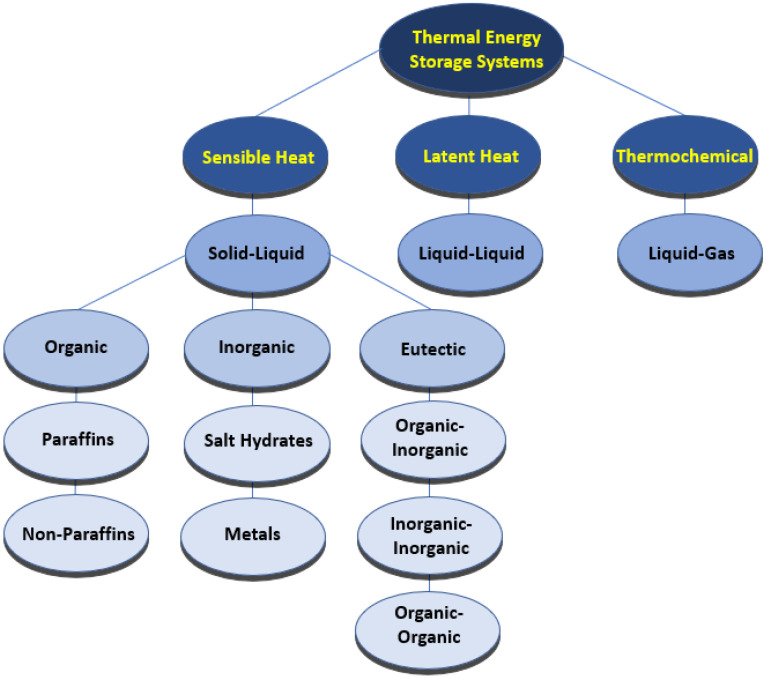
Diagram of the fundamental use strategies of phase change materials in solar thermal energy storage systems.

Many nano-enhanced phase change materials are very suitable for the thermal energy storage technological field. The thermal energy storage systems operating with such materials work according to the passive principle of latent heat energy storage. Such systems allow the thermal management of the storage and release of latent heat. The main limiting parameter is the phase change temperature value. Also, the choice of the most suitable nano-enhanced phase change material for a given system should be based on its preparation method easiness and overall cost, and its improved thermophysical properties. The fundamental interest of researchers was directed to the amelioration of the nano-enhanced phase change materials’ thermal conductivity. In this direction, the authors Lin and Al-Kayiem [44] conducted experimental work on the thermophysical properties of nano-enhanced phase change materials employed in a solar collector. The results indicated that 1% wt. of copper nanoparticles added to the base paraffin wax increased the solar collector efficiency by 1.7% compared to that achieved with only the base phase change material. Also, the authors Natividade et al. [3] studied the efficiency of a solar collector system that includes a parabolic concentrator. Their experiments used a nanofluid composed of water and multilayer graphene at extremely low volume fractions of 0.00045% vol. and 0.00068% vol. to prevent agglomeration and sedimentation. The nanofluid at 0.00045% vol. increased the thermal efficiency by 31% and the nanofluid at 0.00068% increased the thermal efficiency by 76% compared to that obtained with the water alone. The authors Altohamy et al. [148] studied the inclusion of an aqueous alumina nanofluid in a latent heat thermal energy storage system. The investigation team confirmed that the use of the nanofluid had an impact on the solidified mass fraction and charging time of the system, which could decrease considerably. Moreover, the authors found appreciable improvements in the charging rate and the amount of stored energy. The charging time was decreased by up to 30% at 2% vol. as compared to that achieved with pure water. Figure 10 shows the fundamental application strategies of the phase change materials in solar thermal energy storage systems.

### 8.3. Photovoltaic/Thermal Systems

The solar radiation coming onto the surface of photovoltaic/thermal equipment may produce a large amount of electric power. Nonetheless, the absorbed solar radiation enhances the average surface temperature of the photovoltaic module and, consequently, decreases its overall efficiency. It is frequently argued in the literature that the increment in temperature of the photovoltaic panels causes an efficiency drop of nearly 0.5% per degree [149], depending on the used photovoltaic technology. Hence, considering the efficiency and economic feasibility of photovoltaic/thermal systems, it is reasonable to consider that the innovative thermal regulation and beneficial thermophysical characteristics of nano-enhanced phase change materials may be very suitable for this purpose. In this direction, the authors Nada and El-Nagar [150] examined the performance of such materials in the thermal management of integrated photovoltaic modules. The research team noted that the electrical efficiency of the photovoltaic module can be taken as the ratio between its power output and incoming solar radiation. In view of the obtained results, the authors found that the integration into the system of a phase change material of paraffin wax and RT55 from Rubitherm™ and a nano-enhanced phase change material at 2% vol. of alumina nanoparticles resulted in a photovoltaic efficiency increase. The researchers reported that the daily efficiency was enhanced to nearly 5.7% using the phase change material and to approximately 13% employing the nano-enhanced phase change materials. 

### 8.4. Cooling of Electronics

The electronic components and equipment normally generate high heat loads that need to be dissipated. This amount of energy in the form of heat should be removed by cost-effective and efficient cooling routes. Nowadays, the ever-increasing demand for performant and compact electronic devices generates an excessive amount of heat that must be dissipated. Otherwise, the efficiency and lifespan of the electronic equipment will be compromised. With this general purpose, diverse cooling strategies are being devised by the researchers to develop and implement passive and active approaches with the inclusion of nano-enhanced phase change materials. In this sense, the authors Karimi et al. [151] studied the thermal management of a lithium-ion battery using phase change material composites. They employed nanoparticles of copper, silver, and iron oxide with a weight fraction of 2% wt. in a metal matrix and a paraffin mixture as the phase change material. The metal matrix decreased the temperature in the metal container by around 70%, whereas the phase change material system incorporating the copper nanoparticles decreased the container temperature by nearly 60%. Also, the silver and iron oxide nanoparticles incorporated in the phase change material decreased the temperature in the container by half. Moreover, the researcher Temel [152] evaluated the heat transfer capability of the RT-44 paraffin wax with the addition of graphene nanoparticles at different weight fractions in a simulated lithium-ion battery pack under diverse conditions. The author assumed that the battery produces a constant load of heat during its discharge process, with the operating temperature and state of charge left out of the equation. Also, the author stated that the use of the paraffin wax at 7% wt. of nanoparticles under the highest discharge rate of 3.9 W and at 30 °C resulted in an effective thermal protection duration nearly twice as long as that of the pure RT-44 paraffin wax. It resulted also in thermal protection nearly eight times longer than that obtained with natural convection. Additionally, the authors Alimohammadi et al. [153] examined cooling efficiency employing a nano-enhanced phase change material in a chipset. The authors used an inorganic salt-hydrate Mn(NO_3_)_2_ with the incorporation of 1% wt. of iron oxide nanoparticles. Also, three different electronic chipset cooling systems were evaluated: one heat exchanger, one heat exchanger with phase change material, and one heat exchanger with nano-enhanced phase change material. The investigation team reported that the different thermal management systems contributed to lower power consumption by around 4.3%, 8.8%, and 7.3% for each chipset. Additionally, the results showed that the employment of the base phase change material and nano-enhanced phase change material induced a decrease in the chipset average temperature of 14 °C and around 11 °C, respectively, as compared to that achieved when using a common heat sink with natural and forced convection. The researchers Krishna et al. [154] inferred a heat pipe heat transfer performance to be used in the cooling of electronics exploring a nano-enhanced phase change material for thermal energy storage ends. The material was composed of tricosane and different concentrations of alumina nanoparticles. The authors focused on obtaining the distribution of temperature in the charge, discharge, and charge and discharge periods of the condenser, evaporator, and enhanced material. The researchers argued that the use of the mentioned material provoked a temperature drop of nearly 25.8% in the evaporator, which decreased the fan power consumption by 53% compared to a conventional heat pipe. Furthermore, it was observed that the enhanced material was able to store nearly 30% of the energy transferred to the evaporator, thus decreasing the fan power consumption. Also, the authors Fayyaz et al. [155] focused on the combined usage of a nano-enhanced phase change material with distinct heat sink configurations for the cooling of electronic equipment. For this purpose, multi-walled carbon nanotubes were used at 3% wt. and 6% wt. dispersed in RT-42, and there were also finned heat sinks having aluminum square, triangular, and circular pin fins. The authors reported that the circular finned heat sink exhibited the best heat transfer performance with and without the inclusion of the phase change material. Moreover, the research team observed a peak base temperature reduction of 24% in square pin fins with the RT-42. Employing the circular pin finned heat sink and at 6% wt. of carbon nanotubes, the maximum base temperature decreased by around 26%. The authors noted that the circular pin fins with a nano-enhanced phase change material were the most adequate for the base temperature reduction, and all fin configurations with nano-enhanced phase change material decreased the base temperature of the heat sink. The research team also concluded that the inclusion of nanoparticles in the phase change material reduced the discharging period for the phase change material and allowed a faster cooling rate. 

### 8.5. Water Desalination

The currently available water desalination and distillation technological solutions require costly equipment and materials and are not adequate for worldwide large-scale production. Researchers are exploring improved cost–benefit approaches to develop innovative and synergistic methodologies. This is the case of the researchers Chaichan and Kazem [156], who investigated the impact on the overall efficiency of solar distillers of a nano-enhanced phase change material composed of paraffin wax and alumina nanoparticles at concentrations ranging from 0.5% wt. and 3% wt. In their experiments, the authors evaluated the performance of three different solar distillers. The first one was a conventional solar distiller, the second one included paraffin wax, and the last one combined paraffin with alumina nanoparticles. Indeed, the authors found that the utilization of the phase change material enabled an operating period extension of around three hours after sunset. Also, it was reported that the nano-enhanced phase change material increased the distilled amount by nearly 60.5%, achieving a distillery daily production of nearly 3.708 L/m^2^ against production of approximately 2.3 L/m^2^ per day without the use of the nano-enhanced phase change material. Additionally, the researchers Rufuss et al. [80] analyzed diverse solar stills operating with one phase change material and nano-enhanced phase change materials. The first one was paraffin, and the rest were paraffin and weight fractions of 0.3% wt. of titania, copper oxide, and graphene oxide nanoparticles. The authors confirmed that the solar still operating with only pure paraffin had a distillery daily production of around 3.9 L/m^2^, whereas those working with paraffin and the addition of titania, copper oxide, and graphene oxide obtained daily production of approximately 4.9 L/m^2^, 5.3 L/m^2^, and 3.7 L/m^2^, respectively. The investigation team also found that compared to a common solar still, the tested solar stills gave efficiency increments of nearly 23%, 39%, 43%, and 18%, respectively. Furthermore, economic analyses showed that the solar still operating with paraffin and copper oxide nanoparticles was the least costly of all the evaluated solutions.

### 8.6. Engine Exhaust Gas Heat Recovery

The researchers Wilson et al. [157] recovered the heat of exhaust gas from a diesel engine employing a shell-and-tube heat exchanger. The integrated storage tank had a phase change material with the inclusion of copper oxide nanoparticles at a weight fraction of 0.01% wt. Also, the authors studied the variations in temperature of the heat transfer fluid under various engine loads. The obtained results indicated that the full capacity of the engine required a heat consumption of around 4 kW from the system. After testing all loads and conditions, it was confirmed that the thermal effectiveness of the mentioned heat exchanger was over 99%. Nonetheless, the interpretation of this result by the authors was not well elaborated; consequently, this finding should be carefully considered.

### 8.7. Thermal Management of Residential Buildings

The nano-enhanced phase change materials can be applied in residential buildings’ air and water heating using solar energy. The thermal management of residential buildings incorporating nano-enhanced phase change materials can be carried out, among others, with the following practical solutions:

- Solar domestic water heating systems;
- Tankless solar water heaters;
- Photovoltaic/thermal;
- Radiant floors;
- Smart double-glazed windows;
- Thermal batteries;
- Radiators.

**Figure 11:**
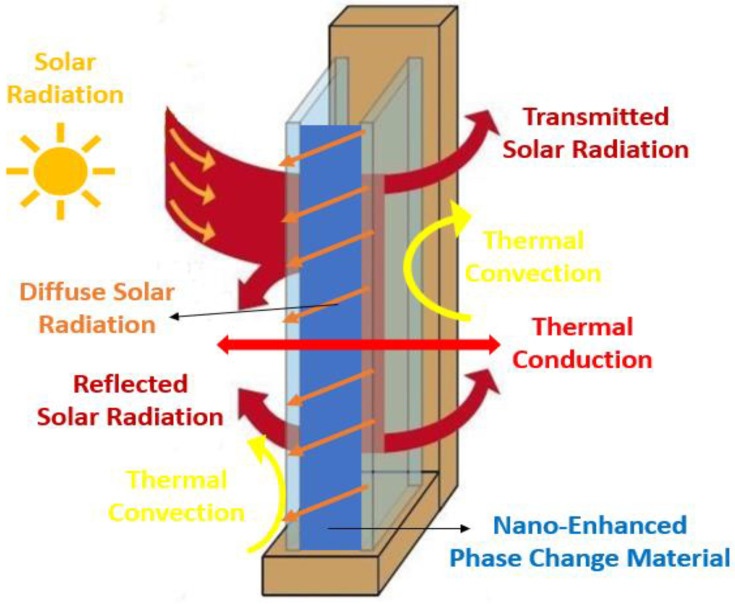
Schematic representation of the thermal mechanisms involved in a double-glazed window with the inclusion of a nano-enhanced phase change material.

The use of solar domestic water heating systems is popular worldwide, but the techno-economic limitations of these systems like heat loss, poor efficiency in cold climates, and costly implementation need to be assessed to improve efficiency. Due to the beneficial features of the phase change materials, several studies were conducted to ameliorate the performance of solar domestic water heating systems with their utilization. In this sense, the author Teamah [158] conducted a review of the application of phase change materials in solar domestic water heating systems and highlighted the possible energy savings associated with the use of phase change materials. In solar domestic water heating systems, the water flows inside a thermally conductive tube or flat plate, which is heated by solar radiation, and a thermally insulated container stores the heated water. In cases where a phase change material is employed, intermediate heat storage is needed to store the extra solar energy during the daytime [159]. According to the literature, metallic and carbon-based nanoparticles have already been evaluated in solar domestic water heating systems because of their superior thermal conductivity [158,159]. Also, the authors Narayanan et al. [160] analyzed a thermal energy storage unit filled with an organic eutectic phase change material and graphite nanostructures. According to the obtained results, the prepared nano-enhanced phase change materials possessed increased thermal conductivity and promising solar energy harvesting ability, without degrading the latent heat of the base phase change material. These benefits led to charging rate enhancements. Also, the researchers Al-Kayiem et al. [161] examined the thermal performance of a solar domestic water heating system with and without the incorporation of a phase change material, and with paraffin and 1% wt. of copper nanoparticles at different inclination angles between 10° and 30°. The researchers reported that the best thermal performance of the system occurred at an inclination of 10°, obtaining approximately 51% and 52% efficiency using the phase change material and nano-enhanced phase change material, respectively. The researchers concluded that there was no appreciable efficiency enhancement using the enhanced material compared to that attained with the phase change material alone. Additionally, the researchers Alshukri et al. [162] investigated the use of paraffin wax having 5% wt. of copper oxide nanoparticles and zinc oxide microparticles in an evacuated tube collector and achieved the highest efficiency enhancement using the copper nanofillers. On the other hand, tankless solar water heaters incorporating phase change materials achieved great attention in the technological area of thermal energy storage because of their effectiveness and inherent compactness to furnish water and space heating in residential building facilities [163]. In this direction, the authors Xie et al. [164] analyzed the techno-economic viability of stearic acid with the addition of coconut shell charcoal to act as a thermal energy storage medium in a domestic tankless solar water heater. The authors demonstrated that the tankless solar water heater could heat the water at night when solar energy was not available. Moreover, the authors Li et al. [56] carried out experimental work to evaluate the applicability of stearic acid in a tankless solar water heater. For this purpose, they used stearic acid at concentrations between 2% wt. and 10% wt. of expandable graphite, and the results demonstrated that the thermal conductivity of the stearic acid at 6% wt. of expandable graphite was almost ten times greater than that attained with pure stearic acid, reaching a value of 2.5 W/mK. The researchers concluded that the addition of expandable graphite improved the heat transfer uniformity, and it represents a very promising option to be applied in tankless solar water heating purposes, given that the thermal storage performance was enhanced with the inclusion of the nano-enhanced phase change material. The most prominent limiting feature of photovoltaic systems is their low energy conversion efficiency derived from their working temperature. This is why nano-enhanced phase change materials were proposed to overcome the adverse effects of high temperatures on the photovoltaic panels. The advantageous features of employing such materials in photovoltaic/thermal systems along with the main studies on the subject were presented in a dedicated section of this paper. Furthermore, radiant floors supply heat directly to the floor and often use for that electric power. These thermally enhanced floors are very thermally efficient since they explore heat sources exhibiting lower temperatures [165]. The critical parameters for developing and implementing radiant floors are the heat transfer ability and average temperature of the surface. Moreover, only a few published experimental and numerical studies examined the efficiency of the radiant floor heating and cooling processes using phase change materials to achieve additional storage capability. Furthermore, the authors Jeon et al. [166] investigated, at a laboratory scale, the effect of adding exfoliated graphene nanoplatelets to paraffin, octadecane, and hexadecane on the thermal conductivity and latent heat of an application in a radiant floor heating system. The thermal conductivity of the octadecane at 5% wt. of exfoliated graphene nanoplatelets was enhanced by 101%. The results showed that adding exfoliated graphene nanoplatelets using octadecane as base phase change material could be a very suitable energy-saving thermal management option in residential buildings. Nonetheless, this study did not examine the nano-enhanced phase change material thermal performance in an actual radiant floor. Furthermore, smart double-glazed windows may be an ideal way to optimize the energy efficiency of residential buildings. Nonetheless, the poor insulation of the windows may lead to excessive cooling/heating charges [167]. Figure 11 schematically illustrates the thermal mechanisms involved in a double-glazed window with the inclusion of a nano-enhanced phase change material.

Studies have been conducted on the thermal performance of double-glazed windows after incorporating phase change materials to enhance their heat storage capacity [168]. In this sense, the researchers Zhang et al. [169] studied the seasonal thermal behavior of a double-glazed window having nanoparticles of alumina, titanium oxide, and zinc oxide dispersed in paraffin. The average temperature values in the interior surfaces were measured in winter and summer to infer the behavior of the diverse nano-enhanced phase change materials. In view of the obtained results, the authors concluded that the inclusion of all the nanoparticles promoted a decrease in energy consumption in the summer season. Nevertheless, there was not any substantial impact on the inner surface in the winter season. A thermal battery or heat battery can store and release thermal energy. Distinct heat sources can be employed for the charging of these systems like photovoltaics and heat pumps. Several authors argued that this type of system provides many benefits such as the heat storage capability ability in the periods where the electricity prices are lower. In this sense, the researchers Violidakis et al. [170] investigated photovoltaic-powered thermal batteries filled with sodium acetate phase change material for the heat and electricity supply in residential buildings. The obtained results demonstrated that a thermal battery charged by a heat pump was the best solution. The comparison of the two cases with a commonly used system proved that the incorporation of a thermal battery enhanced the use of the produced heat. Additionally, nano-enhanced phase change materials can also be employed in the heating and cooling of batteries. For this purpose, the authors Jilte et al. [171] proposed a novel battery thermal management system with the aid of a nano-enhanced phase change material, included in a modified battery exploring two layers of eicosane and Na_2_SO_4_, having melting points of 32.4 °C and 36.4 °C, and alumina nanoparticles. The obtained results demonstrated that the tested enhanced material was a very suitable choice for maintaining the cell temperature below 46 °C. Radiator retrofitting of houses is a challenging concern in reaching the Net Zero target [172]. In this direction, the researchers Sardari et al. [173] examined the thermal behavior of a compact heat storage unit with a composite metal foam phase change material to ameliorate the efficiency of domestic radiators. The unit was fixed to a wall near the radiator to store the waste heat of the back surface of the radiator that was released to the wall. In the cases where the boiler was switched off, the unit released the stored heat, attaining considerable energy savings. The implementation of the nano-enhanced phase change materials in radiators shows some potential, but according to the available literature, this has not yet been conducted. Table 2 summarizes some of the published studies on different applications of nano-enhanced phase change materials.

## 9. Limitations and Prospects for Further Research

The main limitations and prospects for further research studies on nano-enhanced phase change materials for heat transfer ends can be summarized in the following points:

- Further experimental works should be conducted and more accurate preparation routes should be developed for thermal management systems using nano-enhanced phase change materials. In the available published studies, some relevant details are still missing for the sake of the repeatability of results and representative sampling, including the types of base fluids, ideal concentration, optimized synthesis methodologies, safety procedures, and environmental risk assessment.
- Only a few researchers investigating nano-enhanced phase change materials have provided the specific heat capacity determination and discussion. Additionally, the scarce experimental results on the specific heat are not consistent with each other, normally presenting considerable fluctuations. In some of the published studies, the specific heat of the nano-enhanced phase change materials was reported to increase with increasing concentrations of added nanoparticles, and in some others, the opposite trend was verified. Considering this scenario, further investigation studies on specific heat and its influencing factors are recommended.
- Further experimental and numerical works should be conducted to seek further progress in the less-studied areas of application of nano-enhanced phase change materials, such as the cooling of electronics and thermal management of batteries and solar distillers.
- The major concerns about the synthesis, characterization, and employment of the nano-enhanced phase change materials are economic analysis issues. However, the economic viability and investment costs have not been sufficiently assessed and require further in-depth studies.
- It is highly recommended to conduct further analysis on the environmental impact of the nanomaterials incorporated in the nano-enhanced phase change materials to achieve improved knowledge on the subject. Many scientific papers lack the environmental impact in all conceivable stages of the production, use, and final disposal of phase change materials. Also, the available literature does not present extensive guidelines for the safety procedures inherent to the handling, use, and experimental evaluation of these types of materials. Hence, it is strongly suggested to publish the environmental impact evaluation consequences and the description of safety procedures to ensure a safe working environment for the researchers and potential users of the nano-enhanced phase change materials.
- The impact of the added nanoparticles to the base phase change materials on human health is still not yet completely understood. Given this, future experimental works should more intensively explore its potential adverse impacts to establish proper safety guidelines.
- More research studies aiming to develop cost-effective biodegradable nano-enhanced phase change materials should be carried out to produce renewable eco-friendly materials with the ability to be microencapsulated. One potential research path is the analysis of alternative waste-based materials for preparing phase change materials and added nanoparticles.
- Certain issues should be further explored, like the need to lower the cost of the synthesis methods for the nano-enhanced phase change materials and active equipment, and the thermal and long-term stability of these materials to be used in photovoltaic/thermal systems.
- Many of the base phase change materials explored in solar energy storage technology are single-type materials like paraffin wax; consequently, further experimental works involving mixtures of different base phase change materials should be conducted. These works will provide useful insights into the synergistic benefits coming from the improved thermal energy storage capability and stability of such mixtures.
- Most of the studies confirmed that the enhancement route of nano-enhanced phase change materials, incorporating superior thermally conductive nanomaterials, is strongly influenced by their particle shape, size, and constitutive material. Nonetheless, there are considerable discrepancies in the results, which need a better understanding to identify and explain the underlying mechanisms between the nanoparticles and the base phase change materials to infer their impact on the final stability and thermophysical characteristics.
- Diverse research groups should conduct more property measurements to attain the repeatability of the results. Each research team reports their measurements individually, and even though discrepancies are frequently found between the thermophysical properties of the nano-enhanced phase change materials, as they were also often found in the thermophysical properties of the base phase change materials, no vigorous attempts have been made to reproduce those findings. Such an acting mode reduces the accuracy and reliability of the reported results.
- There are limited published data on the thermophysical properties of eutectic mixtures of base phase change materials incorporating nanoparticles. In view of this fact, more research studies are needed on the production and evaluation of nano-enhanced composites containing eutectic base phase change materials.
- Many available techniques to evaluate the stability of nano-enhanced phase change materials are suitable only for small samples, the associated measurement uncertainties are not completely explicit, and there are inconsistent findings in the published data. With these facts in mind, it is suggested to develop, implement, and validate a standardized method to carry out characterization and thermal cycling stability tests on these types of materials.
- Only a small number of available studies have analyzed the heat transfer capability of nano-enhanced phase change materials in real thermal management systems. Though it is vital to determine the thermophysical characteristics of these materials, it is also very important to appraise their thermal behavior within a working real thermal storage system. Also, it is convenient to compare the efficiency of the same real system when using the nano-enhanced phase change materials and when exploring only the base phase change material. Such a procedure will enable a more accurate evaluation of the contribution of the incorporation of the nanoparticles to the thermal performance of the system. Hence, further work on this specific matter is most welcome.

## 10. Conclusions

The main concluding remarks of this overview of nano-enhanced phase change materials are highlighted in the following points:

- The organic phase change materials are very suitable to be applied in solar energy recovery systems because of their intrinsic beneficial features like improved thermal stability and supercooling absence. Hence, it is foreseen that the improved thermal energy storage equipment and systems using phase change materials will have a major role in the research and technological areas of thermal solar energy harvesting and conversion processes.
- The exploration of the nano-enhanced phase change materials greatly enhances the average daily energy storage capability and considerably extends the operating time of solar thermal energy storage systems.
- It was found that most of the nano-enhanced phase change materials’ applications were in the improvement of thermal energy storage systems. The published experimental and numerical studies dealt with the thermal management of energy storage systems, solar collectors, photovoltaic/thermal systems, and engine exhaust gas heat recovery using nano-enhanced phase change materials.
- The average thermal conductivity values when using the nano-enhanced phase change materials were enhanced by up to 100% as compared with those achieved with the traditional thermal fluids themselves.
- The thermal conductivity of the nano-enhanced phase change materials can be adjusted through the different distribution and orientation of the included nanoparticles. Moreover, the addition of porous nanoparticles may aid in the thermal conductivity of the base phase change materials. The porosity of the nanoparticles will decrease the supercooling degree by the great number of active nucleation sites.
- The graphite and graphene-added nanoparticles were extremely good thermal conductivity enhancers for the diverse base phase change materials. Also, the metallic and metal oxide nanoparticles dispersed in the phase change material are good thermal conductivity enhancers, with the induced improvements dependent on their shape, size, and concentration.
- The different types of incorporated nanoparticles cause diverse enhancements in the solidification of the base phase change materials. It is usually observed that the solidification and melting processes of the nano-enhanced phase change materials are influenced by the concentration of the added nanoparticles. However, when a limiting concentration value is surpassed, it can entail some negative results in other thermophysical properties of the phase change material, namely the viscosity. The excessive viscosity of the nano-enhanced phase change materials deteriorates the heat transfer behavior by decreasing the thermal conductivity. Nonetheless, there is the possibility to add solvents to mitigate the viscous effect.
- The latent heat of the phase change materials normally decreases with increasing concentrations of the included nanoparticles, with a few exceptions. The thermophysical properties of the nanoparticles affect latent heat deterioration. Nowadays, there is still no ideal nanoparticle concentration that causes maximum thermal conductivity enhancement and, simultaneously, minor latent heat deterioration.
- The synergistic employment of distinct heat transfer enhancement procedures like the incorporation of nanoparticles, metallic foams, and finned heating surfaces provides an improved heat transport capability of the nano-enhanced phase change materials as compared to that achieved with only one of the heat transfer procedures.
- The combined usage of the nano-enhanced phase change materials and nanofluids is more effective for the thermal management of photovoltaic/thermal cooling than the separate exploration of the nano-enhanced phase change materials. Such a synergetic route normally provides extra heat dissipation to the panels because the heat is extracted in sequence by the phase change material and nanofluid, which are two highly heat-absorbing media. The combined use of the nano-enhanced phase change material and the nanofluid lowers the surface average temperature and improves the temperature uniformity of the photovoltaic panels. Such effects mainly derive from the uniform contact of the nano-enhanced phase change material with the panels.
- The unconverted incident thermal solar energy in photovoltaic/thermal systems can be stored by the nano-enhanced phase change materials under the form of latent heat, which may reduce the average temperature of the panels by more than 30 °C. Additionally, the adoption of a particular nano-enhanced phase change material should be based on many factors including the environment's typical temperature values and latitude, solar irradiation intensity, and wind velocity, among others, given that its effectiveness is more intense during summer than in winter because it absorbs more heat in summer, leading to increased efficiency.
- It was already demonstrated that photovoltaic/thermal systems cooled with two distinct phase change materials at a time are more efficient compared to that provided by only one phase change material because of the improved heat regulation and surface temperature uniformity.
- It was found in the available literature that the efficiency of photovoltaic/thermal systems operating with water can be improved by more than 30% when the nano-enhanced phase change materials are included. Moreover, the general use of nano-enhanced phase change materials may decrease the consumption of non-renewable fossil fuels for electricity production purposes and may considerably reduce the carbon footprint and greenhouse gases.
- The combined use of metallic foams and fins provokes a heat transfer performance enhancement in the thermal management processes of the systems using nano-enhanced phase change materials. The thermal transport network constructed by the foams’ diverse constitutive materials and the effect of the finned surfaces that increase the heat exchange rate between the included nano-enhanced phase change materials and the solar collection system ameliorate the energy harvesting and conversion processes.
- Only very few published studies analyzed the long-term stability of the nano-enhanced phase change materials in terms of thermal conductivity. These studies reported a substantial thermal conductivity decrement after the completion of only a few thermal cycles.

## Figures and Tables

**Table 1:** Experimental works on the thermophysical properties of nano-enhanced phase change materials.

Base Phase Change Materials	Nanoparticles	Remarks on the Thermophysical Properties	Authors	Reference
Sodium Acetate Trihydrate	Copper 10–30 nm	20% thermal conductivity increase at 0.5% wt. Nearly 0.5 °C decrease in the subcooling degree.	Cui et al.	[83]
Erythritol	Aluminum, copper, silica, titanium oxide	8% thermal conductivity increase at copper 2.5% wt. Aluminum had the lowest decrease in the specific heat capacity.	Soni et al.	[84]
Paraffin	Silica 11–14 nm, alumina and iron oxide 20 nm, zinc oxide 50 nm	Nearly 222% increase in the thermal diffusivity at iron oxide 8% wt. The thermal conductivity increased by up to nearly 0.92 w/m.K at alumina 4% wt.	Babapoor and Karimi	[85]
Paraffin	Titanium oxide 160 nm, copper oxide 190 nm, graphene oxide 450 nm	Around 101% thermal conductivity increase at 0.3% wt. graphene oxide. Maximum latent heat capacity increase of nearly 65% with copper oxide. Decrease in the specific heat with all nanoparticles.	Rufuss et al.	[80]
Eicosane	Graphite	4.5-fold thermal conductivity increase at graphite 3.5% wt. 12.5-fold viscosity increase at graphite 3.5% wt.	Srinivasan et al.	[86]
RT50	Copper oxide	Thermal conductivity and viscosity increase. Nearly 11% melting time decrease at 4% wt.	Pahamli et al.	[87]
Paraffin (IGI-1230A)	Helical-form graphene nanofibers, exfoliated graphene nanoplatelets, multi-walled carbon nanotubes	Helical-form graphene nanofibers, exfoliated graphene nanoplatelets did not alter the viscosity. Up to 75% viscosity increase with the multi-walled carbon nanotubes.	Weigand et al.	[88]
RT35	Copper oxide, alumina	2-fold thermal conductivity increase with alumina–copper oxide 75–25%.	Kumar and Krishna	[89]
Paraffin	Hybrid graphene–silver nanostructures	6.7% latent heat increase at 0.3% wt. 90% thermal conductivity increase at 0.3% wt.	Paul et al.	[90]
Polyethylene Glycol	Graphene nanoplatelets	23% thermal conductivity increase at 0.5% wt. 4 K crystallization temperature decrease at 0.5% wt.	Marcos et al.	[91]
Paraffin	Polyaniline and copper oxide	8.2% latent heat capacity increase at 1% wt. polyaniline and 7.8% latent heat capacity increase with copper oxide. Nearly 47% thermal conductivity increase at 1% wt. polyaniline and nearly 64% thermal conductivity increase at 1% polyaniline	George et al.	[92]

**Table 2:** Published studies on the different applications of nano-enhanced phase change materials.

Phase Change Material	Nanoparticles	Application	Remarks	Authors	Reference
Octanoic acid	Expanded graphite	Medical refrigeration and air conditioning	Good thermal conductivity and low melting point	Li et al.	[174]
PDMS	Graphene flakes and graphene foam	Thermal management	Improved latent heat	Zhao et al.	[175]
Palmitic acid	Mullite	Solar energy storing and solar heating	Good shape stability and avoids leakages	Gu et al.	[176]
Ba (OH)_2_ 8H_2_O	Expanded graphite	Industrial Waste Heat Recovery	Enhanced thermal conductivity	Han et al.	[177]
Sodium acetate trihydrate–urea	Expanded graphite	Thermal management	Improved thermal conductivity	Fu et al.	[178]
Oxalic acid dihydrate/glycolic acid binary eutectic	Hydrothermal carbon and polyacrylamide-co-acrylic acid copolymer	Low thermal architectural applications	Improved thermal conductivity	Wang et al.	[179]
Polyethylene glycol	Copper	Building and industrial waste heat recovery	Improved stability and optimal melting temperature	Xu et al.	[180]
1-Octadecanol	Alumina-expanded graphite	Solar energy storage	Supercooling	Gong et al.	[181]
Paraffin	Silica shell/encapsulate	Photovoltaic/thermal systems	The cell temperature decreased by 5 °C and 10 °C Thermal exergy increases of 66% and 208%	Hamada et al.	[182]
OM35	Graphene nanoplatelets	Concentrated photovoltaic cells	The maximum increase in the power output and efficiency were 7% and 6%, respectively, at 0.5% wt.	Sivashankar et al.	[183]
Deionized water	Multi-walled carbon nanotubes	Building cooling and thermal management of intermittently operated electronic devices	The solidification time was reduced by 14–20% at 0.6% wt.	Kumaresan et al.	[184]
Epoxy resin	Aluminum	Building thermal management	Improved latent heat Adequate phase change temperature Good stability	Constantinescu et al.	[185]

## Data Availability

Data sharing is not applicable.

## References

[1] Nourafkan E., Asachi M., Jin H., Wen D., Ahmed W. (2019). Stability and photo-thermal conversion performance of binary nanofluids for solar absorption refrigeration systems. Renew. Energy.

[2] Siddique A.R.M., Kratz F., Mahmud S., Van Heyst B. (2019). Energy Conversion by Nanomaterial-Based Trapezoidal-Shaped Leg of Thermoelectric Generator Considering Convection Heat Transfer Effect. J. Energy Resour. Technol..

[3] Natividade P.S.G., Moura G.D.M., Avallone E., Filho E.B., Gelamo R.V., Gonçalves J.C.D.S.I. (2019). Experimental analysis applied to an evacuated tube solar collector equipped with parabolic concentrator using multilayer graphene-based nanofluids. Renew. Energy.

[4] Salem M., Elsayed M., Abd-Elaziz A., Elshazly K. (2019). Performance enhancement of the photovoltaic cells using Al_2_O_3_/PCM mixture and/or water cooling-techniques. Renew. Energy.

[5] Ding Y., Zheng S., Meng X., Yang D. (2019). Low Salinity Hot Water Injection with Addition of Nanoparticles for Enhancing Heavy Oil Recovery under Reservoir Conditions. Trans. ASME J. Energy Resour. Technol..

[6] Chen Y., Zhu J., Ma H., Chen L., Li R., Jin P. (2019). VO_2_/Nickel-bromine-ionic liquid composite film for thermochromic application. Sol. Energy Mater. Sol. Cells.

[7] Adelekan D.S., Ohunakin O.S., Gill J., Atayero A.A., Diarra C.D., Asuzu E.A. (2019). Experimental performance of a safe charge of LPG refrigerant enhanced with varying concentrations of TiO_2_ nano-lubricant in a domestic refrigerator. J. Therm. Anal. Calorim..

[8] Martín M., Villalba A., Fernandez A.I., Barreneche C. (2019). Development of new nano-enhanced phase change materials (NEPCM) to improve energy efficiency in buildings: Lab-scale characterization. Energy Build..

[9] Ren Q., Xu H., Luo Z. (2019). PCM charging process accelerated with combination of optimized triangle fins and nanoparticles. Int. J. Therm. Sci..

[10] Balakin B.V., Zhdaneev O., Kosinska A., Kutsenko K.V. (2019). Direct absorption solar collector with magnetic nanofluid: CFD model and parametric analysis. Renew. Energy.

[11] Bonab H.B., Javani N. (2019). Investigation and optimization of solar volumetric absorption systems using nanoparticles. Sol. Energy Mater. Sol. Cells.

[12] Kumar G., Mathimani T., Rene E.R., Pugazhendhi A. (2019). Application of nanotechnology in dark fermentation for enhanced biohydrogen production using inorganic nanoparticles. Int. J. Hydrogen Energy.

[13] Zhang J., Qu Z., Maharjan A. (2019). Numerical investigation of coupled optical-electrical-thermal processes for plasmonic solar cells at various angles of incident irradiance. Energy.

[14] Zayed M., Zhao J., Du Y., Kabeel A., Shalaby S. (2019). Factors affecting the thermal performance of the flat plate solar collector using nanofluids: A review. Sol. Energy.

[15] Abdelrazik A.S., Al-Sulaiman F., Saidur R., Ben-Mansour R. (2019). Evaluation of the effects of optical filtration and nanoPCM on the performance of a hybrid photovoltaic-thermal solar collector. Energy Convers. Manag..

[16] Agresti F., Fedele L., Rossi S., Cabaleiro D., Bobbo S., Ischia G., Barison S. (2019). Nano-encapsulated PCM emulsions prepared by a solvent-assisted method for solar applications. Sol. Energy Mater. Sol. Cells.

[17] Rehman T.U., Ali H.M., Janjua M.M., Sajjad U., Yan W.-M. (2019). A critical review on heat transfer augmentation of phase change materials embedded with porous materials/foams. Int. J. Heat Mass Transf..

[18] Sheikholeslami M., Mahian O. (2019). Enhancement of PCM solidification using inorganic nanoparticles and an external magnetic field with application in energy storage systems. J. Clean. Prod..

[19] Punniakodi B.M.S., Senthil R. (2022). Recent developments in nano-enhanced phase change materials for solar thermal storage. Sol. Energy Mater. Sol. Cells.

[20] Kibria M., Anisur M., Mahfuz M., Saidur R., Metselaar I. (2015). A review on thermophysical properties of nanoparticle dispersed phase change materials. Energy Convers. Manag..

[21] Leong K.Y., Abdul Rahman M.R., Gurunathan B.A. (2019). Nano-enhanced phase change materials: A review of thermo-physical properties, applications and challenges. J. Energy Storage.

[22] Teggar M., Arıcı M., Mert M.S., Ajarostaghi S.S.M., Niyas H., Tunçbilek E., Ismail K.A.R., Younsi Z., Benhouia A.T., Mezaache E.H. (2021). A comprehensive review of micro/nano enhanced phase change materials. J. Therm. Anal. Calorim..

[23] De Matteis V., Cannavale A., Martellotta F., Rinaldi R., Calcagnile P., Ferrari F., Ayr U., Fiorito F. (2019). Nano-encapsulation of phase change materials: From design to thermal performance, simulations and toxicological assessment. Energy Build..

[24] Jamekhorshid A., Sadrameli S.M., Farid M. (2014). A review of microencapsulation methods of phase change materials (PCMs) as a thermal energy storage (TES) medium. Renew. Sustain. Energy Rev..

[25] Liu Y., Yang Y. (2017). Investigation of specific heat and latent heat enhancement in hydrate salt based TiO_2_ nanofluid phase change material. Appl. Therm. Eng..

[26] Munyalo J.M., Zhang X., Li Y., Chen Y., Xu X. (2018). Latent heat of fusion prediction for nanofluid based phase change material. Appl. Therm. Eng..

[27] Wang Q., Wei W., Li D., Qi H., Wang F., Arici M. (2019). Experimental investigation of thermal radiative properties of Al_2_O_3_-paraffin nanofluid. Sol. Energy.

[28] Warzoha R.J., Rao A., Weigand R., Fleischer A.S. (2012). Experimental characterization of the thermal diffusivity of paraffin phase change material embedded with herring-bone style graphite nanofibers. Heat Transf. Summer Conf. Am. Soc. Mech. Eng..

[29] Nourani M., Hamdami N., Keramat J., Moheb A., Shahedi M. (2016). Thermal behavior of paraffin-nano-Al_2_O_3_ stabilized by sodium stearoyl lactylate as a stable phase change material with high thermal conductivity. Renew. Energy.

[30] Motahar S., Alemrajabi A.A., Khodabandeh R. (2017). Experimental study on solidification process of a phase change material containing TiO_2_ nanoparticles for thermal energy storage. Energy Convers. Manag..

[31] Wang F., Liu J., Fang X., Zhang Z. (2016). Graphite nanoparticles-dispersed paraffin/water emulsion with enhanced thermal-physical property and photo-thermal performance. Sol. Energy Mater. Sol. Cells.

[32] Chieruzzi M., Cerritelli G.F., Miliozzi A., Kenny J.M., Torre L. (2017). Heat capacity of nanofluids for solar energy storage produced by dispersing oxide nanoparticles in nitrate salt mixture directly at high temperature. Sol. Energy Mater. Sol. Cells.

[33] Ebadi S., Tasnim S.H., Aliabadi A.A., Mahmud S. (2018). Geometry and nanoparticle loading effects on the bio-based nano-PCM filled cylindrical thermal energy storage system. Appl. Therm. Eng..

[34] Salyan S., Suresh S. (2018). Study of thermo-physical properties and cycling stability of d -Mannitol-copper oxide nanocomposites as phase change materials. J. Energy Storage.

[35] Praveen B., Suresh S. (2018). Experimental study on heat transfer performance of neopentyl glycol/CuO composite solid-solid PCM in TES based heat sink. Eng. Sci. Technol. Int. J..

[36] Zeng J.-L., Gan J., Zhu F.-R., Yu S.-B., Xiao Z.-L., Yan W.-P., Zhu L., Liu Z.-Q., Sun L.-X., Cao Z. (2014). Tetradecanol/expanded graphite composite form-stable phase change material for thermal energy storage. Sol. Energy Mater. Sol. Cells.

[37] Mandal S.K., Kumar S., Singh P.K., Mishra S.K., Singh D. (2020). Performance investigation of nanocomposite based solar water heater. Energy.

[38] Kim S., Chang S.J., Chung O., Jeong S.-G., Kim S. (2014). Thermal characteristics of mortar containing hexadecane/xGnP SSPCM and energy storage behaviors of envelopes integrated with enhanced heat storage composites for energy efficient buildings. Energy Build..

[39] Colla L., Fedele L., Mancin S., Danza L., Manca O. (2017). Nano-PCMs for enhanced energy storage and passive cooling applications. Appl. Therm. Eng..

[40] Harish S., Orejon D., Takata Y., Kohno M. (2015). Thermal conductivity enhancement of lauric acid phase change nanocomposite with graphene nanoplatelets. Appl. Therm. Eng..

[41] Sarı A., Biçer A., Hekimoğlu G. (2018). Effects of carbon nanotubes additive on thermal conductivity and thermal energy storage properties of a novel composite phase change material. J. Compos. Mater..

[42] Yadav A., Barman B., Kardam A., Narayanan S.S., Verma A., Jain V. (2017). Thermal properties of nano-graphite-embedded magnesium chloride hexahydrate phase change composites. Energy Environ..

[43] Sharma R., Ganesan P., Tyagi V., Metselaar H., Sandaran S. (2016). Thermal properties and heat storage analysis of palmitic acid-TiO_2_ composite as nano-enhanced organic phase change material (NEOPCM). Appl. Therm. Eng..

[44] Lin S.C., Al-Kayiem H.H. (2016). Evaluation of copper nanoparticles—Paraffin wax compositions for solar thermal energy storage. Sol. Energy.

[45] Sami S., Etesami N. (2017). Improving thermal characteristics and stability of phase change material containing TiO_2_ nanoparticles after thermal cycles for energy storage. Appl. Therm. Eng..

[46] Barreneche C., Mondragon R., Ventura-Espinosa D., Mata J., Cabeza L.F., Fernández A.I., Julia J.E. (2018). Influence of nanoparticle morphology and its dispersion ability regarding thermal properties of water used as phase change material. Appl. Therm. Eng..

[47] Harikrishnan S., Hussain S.I., Devaraju A., Sivasamy P., Kalaiselvam S. (2017). Improved performance of a newly prepared nano-enhanced phase change material for solar energy storage. J. Mech. Sci. Technol..

[48] Mayilvelnathan V., Arasu A.V. (2019). Characterization and thermophysical properties of graphene nanoparticles dispersed erythritol PCM for medium temperature thermal energy storage applications. Thermochim. Acta.

[49] Putra N., Amin M., Kosasih E.A., Luanto R.A., Abdullah N.A. (2017). Characterization of the thermal stability of RT 22 HC/graphene using a thermal cycle method based on thermoelectric methods. Appl. Therm. Eng..

[50] Wang J., Xie H., Guo Z., Guan L., Li Y. (2014). Improved thermal properties of paraffin wax by the addition of TiO_2_ nanoparticles. Appl. Therm. Eng..

[51] Guo S., Liu Q., Zhao J., Jin G., Wang X., Lang Z., He W., Gong Z. (2017). Evaluation and comparison of erythritol-based composites with addition of expanded graphite and carbon nanotubes. Appl. Energy.

[52] Wang J., Xie H., Xin Z. (2009). Thermal properties of paraffin based composites containing multi-walled carbon nanotubes. Thermochim. Acta.

[53] Shaikh S., Lafdi K., Hallinan K. (2008). Carbon nanoadditives to enhance latent energy storage of phase change materials. J. Appl. Phys..

[54] Mohamed N.H., Soliman F.S., El Maghraby H., Moustfa Y. (2017). Thermal conductivity enhancement of treated petroleum waxes, as phase change material, by α nano alumina: Energy storage. Renew. Sustain. Energy Rev..

[55] Israelachvili J.N. (2011). Intermolecular and Surface Forces.

[56] Li C., Zhang B., Xie B., Zhao X., Chen J., Chen Z., Long Y. (2018). Stearic acid/expanded graphite as a composite phase change thermal energy storage material for tankless solar water heater. Sustain. Cities Soc..

[57] Zeng J.L., Cao Z., Yang D.W., Xu F., Sun L.X., Zhang X.F., Zhang L. (2009). Effects of MWNTs on phase change enthalpy and thermal conductivity of a solid-liquid organic PCM. J. Therm. Anal. Calorim..

[58] Meng X., Zhang H., Sun L., Xu F., Jiao Q., Zhao Z., Zhang J., Zhou H., Sawada Y., Liu Y. (2013). Preparation and thermal properties of fatty acids/CNTs composite as shape-stabilized phase change materials. J. Therm. Anal. Calorim..

[59] Wu S., Zhu D., Zhang X., Huang J. (2010). Preparation and Melting/Freezing Characteristics of Cu/Paraffin Nanofluid as Phase-Change Material (PCM). Energy Fuels.

[60] Parameshwaran R., Jayavel R., Kalaiselvam S. (2013). Study on thermal properties of organic ester phase-change material embedded with silver nanoparticles. J. Therm. Anal. Calorim..

[61] Ho C., Gao J. (2009). Preparation and thermophysical properties of nanoparticle-in-paraffin emulsion as phase change material. Int. Commun. Heat Mass Transf..

[62] Yang Y., Luo J., Song G., Liu Y., Tang G. (2014). The experimental exploration of nano-Si_3_N_4_/paraffin on thermal behavior of phase change materials. Thermochim. Acta.

[63] Sun X., Zhang Q., Medina M.A., Lee K.O. (2014). On the Natural Convection Enhancement of Heat Transfer during Phase Transition Processes of Solid-liquid Phase Change Materials (PCMs). Energy Procedia.

[64] Brinkman H.C. (1952). The Viscosity of Concentrated Suspensions and Solutions. J. Chem. Phys..

[65] He Q., Wang S., Tong M., Liu Y. (2012). Experimental study on thermophysical properties of nanofluids as phase-change material (PCM) in low temperature cool storage. Energy Convers. Manag..

[66] Jesumathy S., Udayakumar M., Suresh S. (2011). Experimental study of enhanced heat transfer by addition of CuO nanoparticle. Heat Mass Transf..

[67] Bahiraei F., Fartaj A., Nazri G.-A. (2017). Experimental and numerical investigation on the performance of carbon-based nanoenhanced phase change materials for thermal management applications. Energy Convers. Manag..

[68] Daneshazarian R., Antoun S., Dworkin S.B. (2021). Performance Assessment of Nano-enhanced Phase Change Material for Thermal Storage. Int. J. Heat Mass Transf..

[69] Xu H., Sze J.Y., Romagnoli A., Py X. (2017). Selection of Phase Change Material for Thermal Energy Storage in Solar Air Conditioning Systems. Energy Procedia.

[70] Mjallal I., Farhat H., Hammoud M., Ali S., Assi I. (2018). Improving the Cooling Efficiency of Heat Sinks through the Use of Different Types of Phase Change Materials. Technologies.

[71] Owolabi A.L., Al-Kayiem H.H., Baheta A.T. (2016). Nanoadditives induced enhancement of the thermal properties of paraffin-based nanocomposites for thermal energy storage. Sol. Energy.

[72] Abdelrazik A., Al-Sulaiman F., Saidur R. (2020). Numerical investigation of the effects of the nano-enhanced phase change materials on the thermal and electrical performance of hybrid PV/thermal systems. Energy Convers. Manag..

[73] Zhang Z., Yuan Y., Alelyani S., Cao X., Phelan P.E. (2017). Thermophysical properties enhancement of ternary carbonates with carbon materials for high-temperature thermal energy storage. Sol. Energy.

[74] Wu X., Wu H., Cheng P. (2009). Pressure drop and heat transfer of Al_2_O_3_-H_2_O nanofluids through silicon microchannels. J. Micromech. Microeng..

[75] Safari A., Saidur R., Sulaiman F., Xu Y., Dong J. (2017). A review on supercooling of Phase Change Materials in thermal energy storage systems. Renew. Sustain. Energy Rev..

[76] Kumar K.R.S., Kalaiselvam S. (2017). Experimental investigations on the thermophysical properties of CuO-palmitic acid phase change material for heating applications. J. Therm. Anal. Calorim..

[77] Venkitaraj K.P., Suresh S., Praveen B., Venugopal A., Nair S.C. (2017). Pentaerythritol with alumina nano additives for thermal energy storage applications. J. Energy Storage.

[78] Saeed R.M., Schlegel J., Castano C., Sawafta R. (2018). Preparation and enhanced thermal performance of novel (solid to gel) form-stable eutectic PCM modified by nano-graphene platelets. J. Energy Storage.

[79] Warzoha R.J., Fleischer A.S. (2014). Improved heat recovery from paraffin-based phase change materials due to the presence of percolating graphene networks. Int. J. Heat Mass Transf..

[80] Rufuss D.D.W., Suganthi L., Iniyan S., Davies P. (2018). Effects of nanoparticle-enhanced phase change material (NPCM) on solar still productivity. J. Clean. Prod..

[81] Liu Y.-D., Zhou Y.-G., Tong M.-W., Zhou X.-S. (2009). Experimental study of thermal conductivity and phase change performance of nanofluids PCMs. Microfluid. Nanofluidics.

[82] Hu P., Lu D.-J., Fan X.-Y., Zhou X., Chen Z.-S. (2011). Phase change performance of sodium acetate trihydrate with AlN nanoparticles and CMC. Sol. Energy Mater. Sol. Cells.

[83] Cui W., Yuan Y., Sun L., Cao X., Yang X. (2016). Experimental studies on the supercooling and melting/freezing characteristics of nano-copper/sodium acetate trihydrate composite phase change materials. Renew. Energy.

[84] Soni V., Kumar A., Jain V. (2018). Performance evaluation of nano-enhanced phase change materials during discharge stage in waste heat recovery. Renew. Energy.

[85] Babapoor A., Karimi G. (2015). Thermal properties measurement and heat storage analysis of paraffin nanoparticles composites phase change material: Comparison and optimization. Appl. Therm. Eng..

[86] Srinivasan S., Diallo M.S., Saha S.K., Abass O.A., Sharma A., Balasubramanian G. (2017). Effect of temperature and graphite particle fillers on thermal conductivity and viscosity of phase change material n-eicosane. Int. J. Heat Mass Transf..

[87] Pahamli Y., Hosseini M., Ranjbar A., Bahrampoury R. (2017). Effect of nanoparticle dispersion and inclination angle on melting of PCM in a shell and tube heat exchanger. J. Taiwan Inst. Chem. Eng..

[88] Weigand R., Hess K., Fleischer A.S. (2018). Experimental Analysis of the Impact of Nanoinclusions and Surfactants on the Viscosity of Paraffin-Based Energy Storage Materials. J. Heat Transf..

[89] Kumar M.S., Krishna V.M. (2019). Experimental investigation on performance of hybrid PCM’s on addition of nano particles in thermal energy storage. Mater Today Proc..

[90] Paul J., Samykano M., Pandey A.K., Kadirgama K., Tyagi V.V. (2023). Nano Engineered Paraffin-Based Phase Change Material for Building Thermal Management. Buildings.

[91] Marcos M.A., Cabaleiro D., Guimarey M.J.G., Comuñas M.J.P., Fedele L., Fernández J., Lugo L. (2017). PEG 400-Based Phase Change Materials Nano-Enhanced with Functionalized Graphene Nanoplatelets. Nanomaterials.

[92] George M., Pandey A., Rahim N.A., Tyagi V., Shahabuddin S., Saidur R. (2020). A novel polyaniline (PANI)/ paraffin wax nano composite phase change material: Superior transition heat storage capacity, thermal conductivity and thermal reliability. Sol. Energy.

[93] Zhou D., Yuan J., Zhou Y., Liu Y. (2020). Preparation and characterization of myristic acid/expanded graphite composite phase change materials for thermal energy storage. Sci. Rep..

[94] Chen Y., Zhang Q., Wen X., Yin H., Liu J. (2018). A novel CNT encapsulated phase change material with enhanced thermal conductivity and photo-thermal conversion performance. Sol. Energy Mater. Sol. Cells.

[95] Al-Gebory L., Mengüç M.P. (2018). The effect of pH on particle agglomeration and optical properties of nanoparticle suspensions. J. Quant. Spectrosc. Radiat. Transf..

[96] Mondragón R., Juliá J.E., Cabedo L., Navarrete N. (2018). On the relationship between the specific heat enhancement of salt-based nanofluids and the ionic exchange capacity of nanoparticles. Sci. Rep..

[97] Wang J., Li G., Li T., Zeng M., Sundén B. (2021). Effect of various surfactants on stability and thermophysical properties of nanofluids. J. Therm. Anal. Calorim..

[98] Liu Y., Li X., Hu P., Hu G. (2015). Study on the supercooling degree and nucleation behavior of water-based graphene oxide nanofluids PCM. Int. J. Refrig..

[99] Sandy K., Chantal M., Khalil E.K., Flavia K. Examination and optimization of the design parameters for the thermal hysteresis phenomenon of the phase change material. Proceedings of the 2021 IEEE 3rd International Multidisciplinary Conference on Engineering Technology (IMCET).

[100] Moreles E., Huelsz G., Barrios G. (2018). Hysteresis effects on the thermal performance of building envelope PCM-walls. Build. Simul..

[101] Delcroix B., Kummert M., Daoud A., Bouchard J. (2015). Influence of experimental conditions on measured thermal properties used to model phase change materials. Build. Simul..

[102] Hsu T.-H., Chung C.-H., Chung F.-J., Chang C.-C., Lu M.-C., Chueh Y.-L. (2018). Thermal hysteresis in phase-change materials: Encapsulated metal alloy core-shell microparticles. Nano Energy.

[103] Anand A., Shukla A., Kumar A., Buddhi D., Sharma A. (2021). Cycle test stability and corrosion evaluation of phase change materials used in thermal energy storage systems. J. Energy Storage.

[104] Yang X., Yuan Y., Zhang N., Cao X., Liu C. (2014). Preparation and properties of myristic–palmitic–stearic acid/expanded graphite composites as phase change materials for energy storage. Sol. Energy.

[105] Pielichowska K., Bieda J., Szatkowski P. (2016). Polyurethane/graphite nano-platelet composites for thermal energy storage. Renew. Energy.

[106] Karaipekli A., Sarı A. (2010). Preparation, thermal properties and thermal reliability of eutectic mixtures of fatty acids/expanded vermiculite as novel form-stable composites for energy storage. J. Ind. Eng. Chem..

[107] Mullangi D., Dhavale V., Shalini S., Nandi S., Collins S., Woo T., Kurungot S., Vaidhyanathan R. (2016). Low-Overpotential Electrocatalytic Water Splitting with Noble-Metal-Free Nanoparticles Supported in a sp^3^N-Rich Flexible COF. Adv. Energy Mater..

[108] Mullangi D., Shalini S., Nandi S., Choksi B., Vaidhyanathan R. (2017). Super-hydrophobic covalent organic frameworks for chemical resistant coatings and hydrophobic paper and textile composites. J. Mater. Chem. A.

[109] Evans H.A., Mullangi D., Deng Z., Wang Y., Peh S.B., Wei F., Wang J., Brown C.M., Zhao D., Canepa P. (2022). Aluminum formate, Al(HCOO)_3_: An earth-abundant, scalable, and highly selective material for CO_2_ capture. Sci. Adv..

[110] Khodadadi J., Hosseinizadeh S. (2007). Nanoparticle-enhanced phase change materials (NEPCM) with great potential for improved thermal energy storage. Int. Commun. Heat Mass Transf..

[111] Vajjha R.S., Das D.K. (2009). Experimental determination of thermal conductivity of three nanofluids and development of new correlations. Int. J. Heat Mass Transf..

[112] Gunjo D.G., Jena S.R., Mahanta P., Robi P. (2018). Melting enhancement of a latent heat storage with dispersed Cu, CuO and Al_2_O_3_ nanoparticles for solar thermal application. Renew. Energy.

[113] Iachachene F., Haddad Z., Oztop H.F., Abu-Nada E. (2019). Melting of phase change materials in a trapezoidal cavity: Orientation and nanoparticles effects. J. Mol. Liq..

[114] Gorzin M., Hosseini M.J., Rahimi M., Bahrampoury R. (2019). Nano-enhancement of phase change material in a shell and multi-PCM-tube heat exchanger. J. Energy Storage.

[115] Keshteli A.N., Sheikholeslami M. (2019). Solidification within a wavy triplex-tube heat storage unit utilizing numerical simulation considering Al_2_O_3_ nanoparticles. Phys. A Stat. Mech. Appl..

[116] Haddad Z., Abu-Nada E., Oztop H.F., Mataoui A. (2012). Natural convection in nanofluids: Are the thermophoresis and Brownian motion effects significant in nanofluid heat transfer enhancement?. Int. J. Term. Sci..

[117] Buongiorno J. (2006). Convective Transport in Nanofluids. J. Heat Transfer..

[118] Riahi M.K., Ali M., Addad Y., Abu-Nada E. (2022). Combined Newton–Raphson and streamlines-upwind Petrov–Galerkin it-erations for nanoparticles transport in buoyancy-driven flow. J. Eng. Math..

[119] Amidu M.A., Addad Y., Riahi M.K., Abu-Nada E. (2021). Numerical investigation of nanoparticles slip mechanisms impact on the natural convection heat transfer characteristics of nanofluids in an enclosure. Sci. Rep..

[120] Stock N., Biswas S. (2011). Synthesis of Metal-Organic Frameworks (MOFs): Routes to Various MOF Topologies, Morphologies, and Composites. Chem. Rev..

[121] Farha O.K., Wilmer C.E., Eryazici I., Hauser B.G., Parilla P.A., O’neill K., Sarjeant A.A., Nguyen S.T., Snurr R.Q., Hupp J.T. (2012). Designing Higher Surface Area Metal–Organic Frameworks: Are Triple Bonds Better Than Phenyls?. J. Am. Chem. Soc..

[122] Dey C., Kundu T., Banerjee R. (2011). Reversible phase transformation in proton conducting Strandberg-type POM based metal organic material. Chem. Commun..

[123] Lee Y.-R., Kim J., Ahn W.-S. (2013). Synthesis of metal-organic frameworks: A mini review. Korean J. Chem. Eng..

[124] Kim J., Yang S.-T., Choi S.B., Sim J., Kim J., Ahn W.-S. (2011). Control of catenation in CuTATB-n metal–organic frameworks by sonochemical synthesis and its effect on CO_2_ adsorption. J. Mater. Chem..

[125] Khan N.A., Haque E., Jhung S.H. (2010). Rapid syntheses of a metal–organic framework material Cu_3_(BTC)_2_(H_2_O)_3_ under microwave: A quantitative analysis of accelerated syntheses. Phys. Chem. Chem. Phys..

[126] Moh P.Y., Cubillas P., Anderson M.W., Attfield M.P. (2011). Revelation of the Molecular Assembly of the Nanoporous Metal Organic Framework ZIF-8. J. Am. Chem. Soc..

[127] Radhakrishnan L., Reboul J., Furukawa S., Srinivasu P., Kitagawa S., Yamauchi Y. (2011). Preparation of Microporous Carbon Fibers through Carbonization of Al-Based Porous Coordination Polymer (Al-PCP) with Furfuryl Alcohol. Chem. Mater..

[128] Xu X., Cao R., Jeong S., Cho J. (2012). Spindle-like Mesoporous α-Fe_2_O_3_ Anode Material Prepared from MOF Template for High-Rate Lithium Batteries. Nano Lett..

[129] Ma S., Goenaga G.A., Call A.V., Liu D.-J. (2011). Cobalt Imidazolate Framework as Precursor for Oxygen Reduction Reaction Electrocatalysts. Chem. Eur. J..

[130] Maity R., Chakraborty D., Nandi S., Yadav A.K., Mullangi D., Vinod C.P., Vaidhyanathan R. (2019). Aqueous-Phase Differentiation and Speciation of Fe^3+^ and Fe^2+^ Using Water-Stable Photoluminescent Lanthanide-Based Metal–Organic Framework. ACS Appl. Nano Mater..

[131] Huang N., Zhai L., Coupry D.E., Addicoat M.A., Okushita K., Nishimura K., Heine T., Jiang D. (2016). Multiple-component covalent organic frameworks. Nat. Commun..

[132] Guan X., Chen F., Fang Q., Qiu S. (2020). Design and applications of three dimensional covalent organic frameworks. Chem. Soc. Rev..

[133] Mullangi D., Chakraborty D., Pradeep A., Koshti V., Vinod C.P., Panja S., Nair S., Vaidhyanathan R. (2018). Highly Stable COF-Supported Co/Co(OH)_2_ Nanoparticles Heterogeneous Catalyst for Reduction of Nitrile/Nitro Compounds under Mild Conditions. Small.

[134] Chakraborty D., Mullangi D., Chandran C., Vaidhyanathan R. (2022). Nanopores of a Covalent Organic Framework: A Customizable Vessel for Organocatalysis. ACS Omega.

[135] Li J., Lu W., Zeng Y., Luo Z. (2014). Simultaneous enhancement of latent heat and thermal conductivity of docosane-based phase change material in the presence of spongy graphene. Sol. Energy Mater. Sol. Cells.

[136] Al-Jethelah M., Ebadi S., Venkateshwar K., Tasnim S., Mahmud S., Dutta A. (2018). Charging nanoparticle enhanced bio-based PCM in open cell metallic foams: An experimental investigation. Appl. Therm. Eng..

[137] Masoumi H., Khoshkhoo R.H., Mirfendereski S.M. (2019). Modification of physical and thermal characteristics of stearic acid as a phase change materials using TiO_2_-nanoparticles. Thermochim. Acta.

[138] Sivasamy P., Harikrishnan S., Hussain S.I., Kalaiselvam S., Babu L.G. (2019). Improved thermal characteristics of Ag nanoparticles dispersed myristic acid as composite for low temperature thermal energy storage. Mater. Res. Express.

[139] Song S., Qiu F., Zhu W., Guo Y., Zhang Y., Ju Y., Feng R., Liu Y., Chen Z., Zhou J. (2019). Polyethylene glycol/halloysite@Ag nanocomposite PCM for thermal energy storage: Simultaneously high latent heat and enhanced thermal conductivity. Sol. Energy Mater. Sol. Cells.

[140] Li T., Wu D., He F., Wang R. (2017). Experimental investigation on copper foam/hydrated salt composite phase change material for thermal energy storage. Int. J. Heat Mass Transf..

[141] Xiao Q., Zhang M., Fan J., Li L., Xu T., Yuan W. (2019). Thermal conductivity enhancement of hydrated salt phase change materials employing copper foam as the supporting material. Sol. Energy Mater. Sol. Cells.

[142] Sheng N., Dong K., Zhu C., Akiyama T., Nomura T. (2019). Thermal conductivity enhancement of erythritol phase change material with percolated aluminum filler. Mater. Chem. Phys..

[143] Wei H., Li X. (2017). Preparation and characterization of a lauric-myristic-stearic acid/Al_2_O_3_-loaded expanded vermiculite composite phase change material with enhanced thermal conductivity. Sol. Energy Mater. Sol. Cells.

[144] Singh D., Salyan S. Thermo-chemical stability of nano composite myo- inositol for solar thermal energy storage. Proceedings of the International Conference on Recent Trends in Engineering & Technology, CUSAT.

[145] Abdelrahman H., Wahba M., Refaey H., Moawad M., Berbish N. (2019). Performance enhancement of photovoltaic cells by changing configuration and using PCM (RT35HC) with nanoparticles Al_2_O_3_. Sol. Energy.

[146] Zarma I., Ahmed M.A., Ookawara S. (2019). Enhancing the performance of concentrator photovoltaic systems using Nanoparticle-phase change material heat sinks. Energy Convers. Manag..

[147] Zeng Y., Fan L.W., Xiao Y.Q., Yu Z.T., Cen K.F. (2013). An experimental investigation of melting of nanoparticle-enhanced phase change materials (NePCMs) in a bottom-heated vertical cylindrical cavity. Int. J. Heat Mass Transf..

[148] Altohamy A.A., Rabbo M.A., Sakr R., Attia A.A. (2015). Effect of water based Al_2_O_3_ nanoparticle PCM on cool storage performance. Appl. Therm. Eng..

[149] Hasan A., McCormack S., Huang M., Norton B. (2010). Evaluation of phase change materials for thermal regulation enhancement of building integrated photovoltaics. Sol. Energy.

[150] Nada S., El-Nagar D. (2018). Possibility of using PCMs in temperature control and performance enhancements of free stand and building integrated PV modules. Renew. Energy.

[151] Karimi G., Azizi M., Babapoor A. (2016). Experimental study of a cylindrical lithium ion battery thermal management using phase change material composites. J. Energy Storage.

[152] Temel U.N. (2019). Passive thermal management of a simulated battery pack at different climate conditions. Appl. Therm. Eng..

[153] Alimohammadi M., Aghli Y., Alavi E.S., Sardarabadi M., Passandideh-Fard M. (2017). Experimental investigation of the effects of using nano/phase change materials (NPCM) as coolant of electronic chipsets, under free and forced convection. Appl. Therm. Eng..

[154] Krishna J., Kishore P., Solomon A.B. (2017). Heat pipe with nano enhanced-PCM for electronic cooling application. Exp. Therm. Fluid Sci..

[155] Fayyaz H., Hussain A., Ali I., Shahid H., Ali H.M. (2022). Experimental Analysis of Nano-Enhanced Phase-Change Material with Different Configurations of Heat Sinks. Materials.

[156] Chaichan M.T., Kazem H.A. (2018). Single slope solar distillator productivity improvement using phase change material and Al_2_O_3_ nanoparticle. Sol. Energy.

[157] Wilson J., Singh A., Singh A., Ganapathy S. (2017). Waste heat recovery from diesel engine using custom designed heat exchanger and thermal storage system with nanoenhanced phase change material. Therm. Sci..

[158] Teamah H. (2021). Comprehensive review of the application of phase change materials in residential heating applications. Alex. Eng. J..

[159] Qiu L., Ouyang Y., Feng Y., Zhang X. (2019). Review on micro/nano phase change materials for solar thermal applications. Renew. Energy.

[160] Narayanan S.S., Kardam A., Kumar V., Bhardwaj N., Madhwal D., Shukla P., Kumar A., Verma A., Jain V. (2017). Development of sunlight-driven eutectic phase change material nanocomposite for applications in solar water heating. Resour. Technol..

[161] Al-Kayiem H.H., Lin S.C. (2014). Performance evaluation of a solar water heater integrated with a PCM nanocomposite TES at various inclinations. Sol. Energy.

[162] Alshukri M.J., Eidan A.A., Najim S.I. (2021). The influence of integrated Micro-ZnO and Nano-CuO particles/paraffin wax as a thermal booster on the performance of heat pipe evacuated solar tube collector. J. Energy Storage.

[163] Der J.P., Kostiuk L.W., McDonald A.G. (2017). Analysis of the performance of a tankless water heating combo system: Simultaneous space heating and domestic hot water operation. Energy Build..

[164] Xie B., Li C., Zhang B., Yang L., Xiao G., Chen J. (2020). Evaluation of stearic acid/coconut shell charcoal composite phase change thermal energy storage materials for tankless solar water heater. Energy Built Environ..

[165] Babiak J., Olesen B.W., Petras D. (2009). Low Temperature Heating and High Temperature Cooling: Embedded Water Based Surface Heating and Cooling Systems. Rehva.

[166] Jeon J., Jeong S.-G., Lee J.-H., Seo J., Kim S. (2012). High thermal performance composite PCMs loading xGnP for application to building using radiant floor heating system. Sol. Energy Mater. Sol. Cells.

[167] Li D., Wang B., Li Q., Liu C., Arici M., Wu Y. (2019). A numerical model to investigate non-gray photothermal characteristics of paraffin-containing glazed windows. Sol. Energy.

[168] Goia F., Perino M., Haase M. (2012). A numerical model to evaluate the thermal behaviour of PCM glazing system configurations. Energy Build..

[169] Zhang G., Wang Z., Li D., Wu Y., Arıcı M. (2020). Seasonal thermal performance analysis of glazed window filled with paraffin including various nanoparticles. Int. J. Energy Res..

[170] Violidakis I., Atsonios K., Iliadis P., Nikolopoulos N. (2020). Dynamic modeling and energy analysis of renewable heating and electricity systems at residential buildings using phase change material based heat storage technologies. J. Energy Storage.

[171] Jilte R., Afzal A., Panchal S. (2021). A novel battery thermal management system using nano-enhanced phase change materials. Energy.

[172] Ferrante A., Semprini G. (2011). Building energy retrofitting in urban areas. Procedia Eng..

[173] Sardari P.T., Babaei-Mahani R., Giddings D., Yasseri S., A Moghimi M., Bahai H. (2020). Energy recovery from domestic radiators using a compact composite metal Foam/PCM latent heat storage. J. Clean. Prod..

[174] Li Y., Zhang X., Munyalo J.M., Tian Z., Ji J. (2019). Preparation and thermophysical properties of low temperature composite phase change material octanoic-lauric acid/expanded graphite. J. Mol. Liq..

[175] Zhao Y.-H., Zhang Y.-F., Bai S.-L. (2016). High thermal conductivity of flexible polymer composites due to synergistic effect of multilayer graphene flakes and graphene foam. Compos. Part A Appl. Sci. Manuf..

[176] Gu X., Liu P., Bian L., He H. (2019). Enhanced thermal conductivity of palmitic acid/mullite phase change composite with graphite powder for thermal energy storage. Renew. Energy.

[177] Han X., Zhang X., Hua W., Yuan W., Jia X., Wang Z.F. (2019). Preparation and application of composite EG/Ba(OH)_2_ ·8H_2_O form-stable phase change material for solar thermal storage. Int. J. Energy Res..

[178] Fu W., Zou T., Liang X., Wang S., Gao X., Zhang Z., Fang Y. (2018). Thermal properties and thermal conductivity enhancement of composite phase change material using sodium acetate trihydrate–urea/expanded graphite for radiant floor heating system. Appl. Therm. Eng..

[179] Wang J., Han W., Ge C., Guan H., Yang H., Zhang X. (2019). Form-stable oxalic acid dihydrate/glycolic acid-based composite PCMs for thermal energy storage. Renew. Energy.

[180] Xu S., Zhang X., Huang Z., Liu Y., Fang M., Wu X., Min X. (2018). Thermal conductivity enhanced polyethylene glycol/expanded perlite shape-stabilized composite phase change materials with Cu powder for thermal energy storage. Mater. Res. Express.

[181] Gong S., Cheng X., Li Y., Shi D., Wang X., Zhong H. (2019). Enhancement of ceramic foam modified hierarchical Al_2_O_3_@expanded graphite on thermal properties of 1-octadecanol phase change materials. J. Energy Storage.

[182] Hamada A.T., Sharaf O.Z., Orhan M.F. (2023). A novel photovoltaic/thermal (PV/T) solar collector based on a multi-functional nano-encapsulated phase-change material (nano-ePCM) dispersion. Energy Convers. Manag..

[183] Sivashankar M., Selvam C., Manikandan S., Harish S. (2020). Performance improvement in concentrated photovoltaics using nano-enhanced phase change material with graphene nanoplatelets. Energy.

[184] Kumaresan V., Velraj R., Das S.K. (2012). The effect of carbon nanotubes in enhancing the thermal transport properties of PCM during solidification. Heat Mass Transf..

[185] Constantinescu M., Dumitrache L., Constantinescu D., Anghel E., Popa V., Stoica A., Olteanu M. (2010). Latent heat nano composite building materials. Eur. Polym. J..

